# Comparative metagenomic analysis from Sundarbans ecosystems advances our understanding of microbial communities and their functional roles

**DOI:** 10.1038/s41598-024-67240-1

**Published:** 2024-07-13

**Authors:** Basanta Kumar Das, Hirak Jyoti Chakraborty, Vikash Kumar, Ajaya Kumar Rout, Biswanath Patra, Sanjoy Kumar Das, Bijay Kumar Behera

**Affiliations:** ICAR-Central Inland Fisheries Research Institute, Barrackpore, Kolkata, West Bengal 700120 India

**Keywords:** Sundarbans, Metagenomics, Microbial communities, Probiotics, Bioremediation, Microbial communities, Environmental microbiology

## Abstract

The Sundarbans mangrove, located at the mouth of the Ganges and Brahmaputra Rivers, is the world’s largest tidal mangrove forest. These mangroves are also one of the most striking sources of microbial diversity, essential in productivity, conservation, nutrient cycling, and rehabilitation. Hence, the main objective of this study was to use metagenome analysis and provide detailed insight into microbial communities and their functional roles in the Sundarbans mangrove ecosystem. A comparative analysis was also done with a non-mangrove region of the Sundarbans ecosystem to assess the capability of the environmental parameters to explain the variation in microbial community composition. The study found several dominant bacteria, viz., *Alphaproteobacteria*, *Actinomycetota*, *Bacilli*, *Clostridia*, *Desulfobacterota*, *Gammaproteobacteria*, and *Nitrospira,* from the mangrove region. The mangrove sampling site reports several salt-tolerant bacteria like *Alkalibacillus haloalkaliphilus*, *Halomonas anticariensis*, and *Salinivibrio socompensis*. We found some probiotic species, viz., *Bacillus clausii, Lactobacillus curvatus, Vibrio mediterranei* and* Vibrio fluvialis,* from the Sundarbans mangrove. Nitrifying bacteria in Sundarbans soils were *Nitrococcus mobilis*, *Nitrosococcus oceani, Nitrosomonas halophila, Nitrospirade fluvii,* and others. Methanogenic archaea, viz., *Methanoculleus marisnigri*, *Methanobrevibacter gottschalkii,* and *Methanolacinia petrolearia,* were highly abundant in the mangroves as compared to the non-mangrove soils. The identified methanotrophic bacterial species, viz., *Methylobacter tundripaludum, Methylococcus capsulatus, Methylophaga thiooxydans,* and *Methylosarcina lacus* are expected to play a significant role in the degradation of methane in mangrove soil. Among the bioremediation bacterial species identified, *Pseudomonas alcaligenes*, *Pseudomonas mendocina*, *Paracoccus denitrificans,* and *Shewanella putrefaciens* play a significant role in the remediation of environmental pollution. Overall, our study shows for the first time that the Sundarbans, the largest mangrove ecosystem in the world, has a wide range of methanogenic archaea, methanotrophs, pathogenic, salt-tolerant, probiotic, nitrifying, and bioremediation bacteria.

## Introduction

Sundarbans, the largest mangrove forest in the world, is situated in the joint delta of the Ganges, Brahmaputra and Meghna rivers in the Bay of Bengal. This UNESCO World Heritage site comprises the Indian state of West Bengal and southwest Bangladesh. The livelihood and well-being of millions living in and around Sundarbans depend on its status and ecological services^1,2^. Despite its high environmental and economic values, Sundarbans is seriously threatened by different anthropogenic activities. Since the early nineteenth century, the landscapes of Sundarbans have also been changing due to saline and freshwater imbalances. The water quality of this ecosystem is primarily affected by sewage pollutants from industries located upstream and in urban areas of West Bengal. Sewage entering coastal water contains diverse chemical and microbiological pollutants and a wide variety of organic and inorganic wastes, driving changes in ecological and physiological health^3,4^.

Microbial communities of mangroves are responsible for nutrient cycling and play a vital role in the productivity, conservation and rehabilitation of mangrove ecosystems^5,6^. Moreover, the fluctuating physicochemical conditions of flooding, salinity, light, and temperature^7^ in mangrove ecosystems have led to the evolution of microorganisms, making them recognized hotspots of microbial diversity. Therefore, understanding the microbial communities and their interaction with the mangrove ecosystem would be crucial in predicting changes in service-provisioning^8,9^. Several recent studies described the composition of the microbial community of surface sediments and water in Indian Sundarban mangrove areas. Surface sediments in this area are dominated by Deltaproteobacteria followed by Gammaproteobacteria, Alphaproteobacteria, Betaproteobacteria, and Epsilonproteobacteria under phylum Pseudomonadota. Abundant bacterial orders are Desulfobacterales, Desulfuromonadales, Myxococcales, and Bdellovibrionales. In contrast, bacterioplankton communities in the water of this region were found to be abundant with Gammaproteobacteria and Alphaproteobacteria. At the family level, the dominancy of Hyphomicrobiaceae, Rhodobacteraceae, Pseudomonadaceae, Erythrobacteraceae, Kordiimonadaceae, Hyphomonadaceae, and Ruminococcaceae was observed^10,11^.

Cellulases, hemicellulases, carbohydrate-binding domains, dockerins, and cohesins were identified using metagenomic sequence analysis with a higher possibility of finding cultivable fungi and bacteria for biomass decomposition and production of biofuels. The metagenomics study showed a similar prevalence of bacterial phyla in Malaysian mangrove system and identified Pseudomonadota (53.5% and 55.1%), followed by Bacillota (11.5% and 10.4%), Bacteroidota (7.4% and 6.2%), Chloroflexota (5.4% and 5.2%), Actinomycetota (4.5% and 5.0%), Planctomycetota (3.7% and 4.3%) and Cyanobacteria (3.2% and 3.3%)^12,13^. Remarkably, the *Bacillus* strains isolated from the mangrove ecosystems in peninsular Malaysia were reported to degrade microplastic elements. *Bacillus cereus* and *Bacillus gottheilii* degraded the microplastic material in the microplastic-contaminated environment, depicting remediation’s role^14^. In Thailand’s mangrove sediment, five cultivatable bacteria (*Mycobacterium* spp. PO1 and PO2, *Novosphingobium pentaromativorans* PY1, *Ochrobactrum* sp. PW1, and *Bacillus* sp. FW1) were meticulously identified^15^. The artificially assembled consortium comprising Mycobacterium and the other three strains (PY1, PW1, and FW1) demonstrated a degradation rate for pyrene three times higher than that of standalone Mycobacterium; this can be attributed to the collaborative interactions within the bacterial blend. Metagenomic analysis from river Ganga and Yamuna sediment samples identified bacteriophages, probiotics and bioremediation bacteria^16,17^. The AMR (antibiotic resistance genes) were reported from the river Yamuna sediment through metagenome analysis^8^. Metagenomics has become the best way to learn about the detailed taxonomic profiles and functions of bacterial, viral, and planktonic communities and how they interact with each other^18^.

Methanotrophs are essential microbial communities in mangrove ecosystems, having unique properties to absorb and metabolize methane gas, ultimately reducing greenhouse emissions from the atmosphere^19^. Methylobacter and Methylosarcina were found to be the essential active methanotrophs in dwarf mangrove soils, whereas Methylomonas was found to be abundant in mangrove soils under tidal influence. The investigation of methane (CH_4_) oxidation and the composition of the methanotrophic community in an unspoiled New Zealand beech forest demonstrated a diverse methanotrophic population with a marginally increased presence of type II methanotrophs^9^. The Sundarbans soil harbours various species of probiotic bacteria that have potential applications in aquaculture.

Few studies have examined the microbial communities in the Indian Sundarbans mangrove water, mainly restricted to an island (Sagar Island) and other estuaries of Mooriganga, Thakuran, Matla, and Harinbhanga. Therefore, the major conclusions of these studies were made based on a limited number of samples. Moreover, little effort has been made to perform a comparative analysis of microbial communities from Sundarbans mangroves and non-mangrove ecosystems^20,21^. Therefore, our knowledge of those communities and information on how environmental parameters control them is limited.

The objectives of this study were to characterize the taxonomic relative abundance and biodiversity of Sundarbans mangrove and non-mangrove ecosystems through metagenome analysis. We also explored the environmental determinants contributing to the variation of their microbial communities. A metagenomic database consisting of microbial taxa by geographic site was constructed to corroborate differences or similarities between bacterial communities’ structure among mangrove and non-mangrove ecosystems with different physicochemical characteristics. Another aim was to assess bacterial species with the highest potential for bioremediation, including probiotic species, nitrifying bacteria, and methanogenic and methanotrophic species. This study will provide baseline knowledge on the microbial ecology of the Sundarbans ecosystems and serve as a baseline for monitoring programs and predicting changes at impacted sites.

## Material and methods

### Sample collection

**Figure 1:**
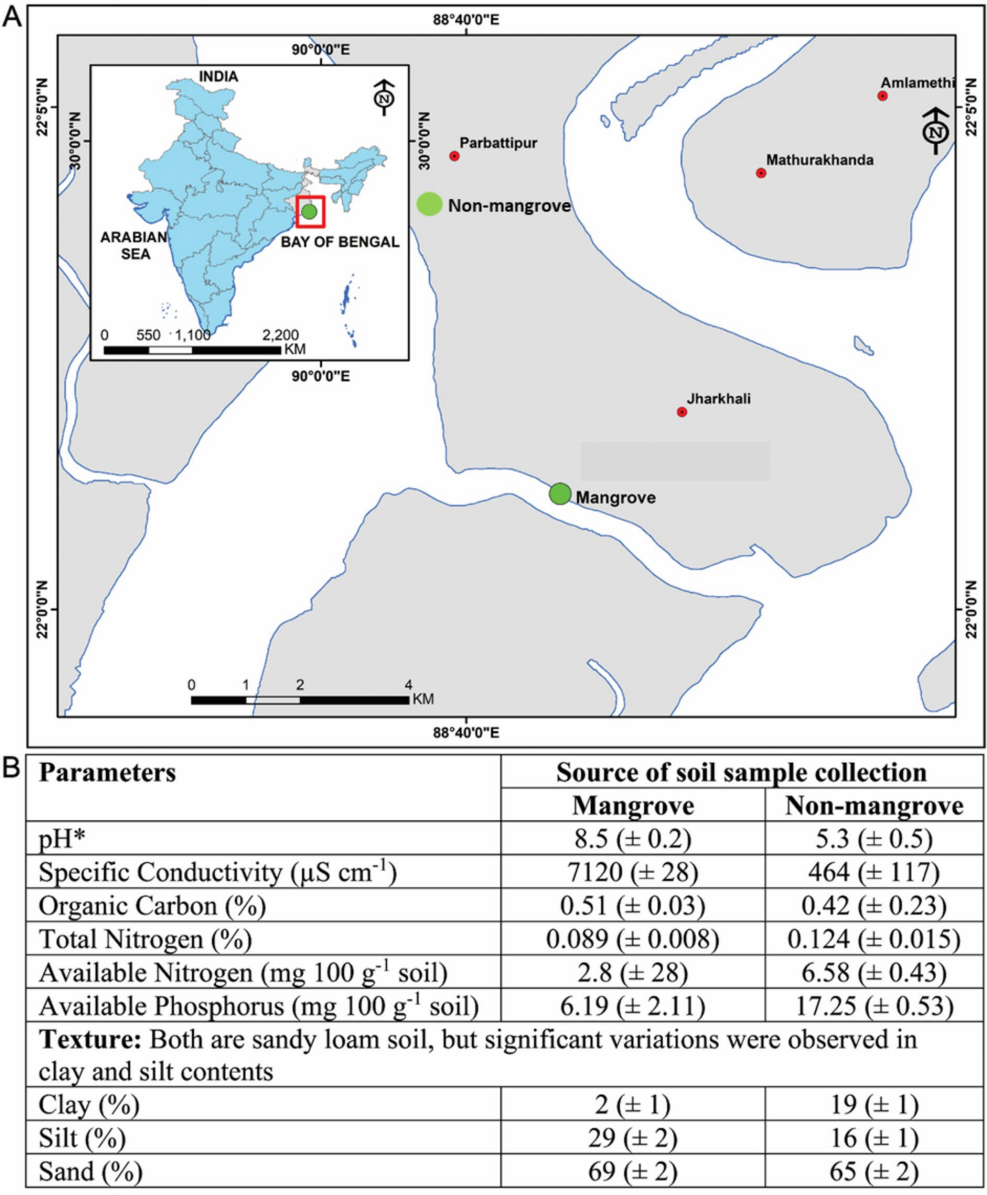
(**A**) Map showing the soil sampling sites. Soil was collected from mangrove and Non-mangrove areas of Sundarban, West Bengal, India. The map of soil collection sites was prepared using the ArcGIS 10.2.1 platform. (**B**) Properties of physical and chemical parameters of soil samples (ArcGIS- https://www.arcgis.com/index.html).

The mangrove sediment samples were collected from Jharkhali (9 g/L, Sundarbans regions), Basanti block, South 24 Paraganas, West Bengal, India within planation areas of tidal influence bounded by 22° 01′ 09″ N to 88° 40′ 56″ E latitude and longitudes. While, the non-mangroves soil samples were collected from a harvested paddy field of Sonatikari (1 g/ L), South 24 Paraganas, West Bengal, India (22° 01′ 24.0″ N 88° 30′ 19.1″ E) (Fig. 1A).

Sediment samples were obtained utilizing a Van Veen grab from the surface benthic layer (upper 20 cm at the water column interface), indicating a more recent microbial community in the sediment–water interface’s surface layer. One sediment grab per location was gathered and subjected to particle sieve analysis for grain size determination. From the twelve geographical sites of both mangrove and non-mangrove ecosystems, a total of 9 sediment samples from each ecosystem were combined and examined. Around 30 g of sediment from the grab was moved to a sterile 50 mL Falcon™ tube. The tubes containing the samples were quickly placed in an ice-filled container on board until they were transported to the laboratory for further analysis.

### Physical and chemical properties of soil sample

The evaluation of physiochemical properties required the quantification of sediment samples. For the assessment of sediment physiochemical quality, initial preparation involved drying the samples in a shaded area at room temperature, followed by grinding using a wooden hammer. Subsequently, the samples were sieved through a No. 10 mesh sieve (2 mm) and preserved in plastic packets. The soil samples were analyzed for pH (VWR International, USA), specific conductivity (µS cm^−1^) (BioRad, USA), organic carbon (%), total nitrogen (%) (Borosil, India), available nitrogen (mg 100 g^−1^ soil), available phosphorus (mg 100 g^−1^ soil) (BioRad, USA), clay (%), silt (%), sand (%) as per the standard methods (APHA 2012).

Later, the pooled samples were air-dried, ground, and sieved through 10 meshe before going for laboratory analyses. We used standard probes to measure conductivity and pH in 1:2.5 soil–water suspensions. Walkley and Black^22^ method was followed for the estimation of organic carbon where, after oxidation of soil organic matter by K_2_Cr_2_O_7_ and concentrated H_2_SO_4_, unconsumed K_2_Cr_2_O_7_ was back titrated against standard ferrous ammonium sulphate solution^23^. Kjeldahl’s method estimated the total and available nitrogen^24^ and alkaline-permanganate method, respectively^25^. Available phosphorus was measured by a spectroscopic method using Olsen’s extraction^26^; ascorbic acid was used for blue colour development. Soil texture was determined by mechanical analysis following the Bouyoucos Hydrometer method^27^.

### Genomic DNA extraction

The conventional phenol–chloroform extraction method isolated the DNA from pooled soil samples. Approximately 200 ng of isolated DNA was run on 0.8% Agarose gel at 120 V for about 60 min or until the sample reached 3/4th of the total gel. One μl of each sample was fed into the Nanodrop 2000 to calculate the A260/280 ratio, and the Qubit 3.0 Fluorometer was used to measure the amount of genomic DNA present. Genomic DNA from high-quality soil was used to make next-generation libraries.

### Library construction and quality control

The TruSeq Nano DNA Library Preparation kit from Illumina was utilized to create the paired-end sequencing libraries for samples that passed quality control. The end-repair mechanism was employed to process the fragments, thus ensuring a minimal occurrence of chimera (concatenated template) formation. After ligation, AMPure XP beads were used to select the size of the products. Subsequently, PCR amplification with the index primer followed the TrueSeq DNA Nano Kit-Illumina protocol. Lastly, the PCR-enriched libraries were analyzed using the 4200 Tape Station system’s High Sensitivity D1000 Screen Tape (Agilent Technologies) in compliance with the manufacturer’s guidelines.

### Cluster generation and sequencing

After determining the Qubit concentration and the average peak size from the Agilent Tape Station profile, the Pair-End Illumina library was placed onto NextSeq 500 for cluster generation and sequencing. Pair-end sequencing enables sequencing template fragments in both forward and reverse directions, leading to enhanced sequencing coverage across many organisms. The average fragment size distributions for mangrove and non-mangrove groups were 448 bp and 419 bp, respectively. Library preparation involved attaching ligation adapters made for targeting short paired ends (2 × 150 bp). Large insert libraries were subsequently developed to accommodate the diversity in samples and optimize high-quality assembly for all microbial taxa.

### Trimming, *Denovo* Assembly and gene prediction of metagenome data

The raw data sequence underwent processing to procure high-quality, clean reads. Trimmomatic v0.35 was employed to eliminate adapter sequences, ambiguous reads (those with over 5% unknown nucleotides “N”), and low-grade sequences (reads exceeding ten quality thresholds (QV) with a phred score below 20)^28^. After trimming, a minimum length of 100 nts was allowed (http://www.clcbio.com/). After removing the adapter and low-quality sequences from the raw data, 40,877,196 (2 × 150 bp) and 34.413,814 (2 × 150 bp) high-quality reads were obtained for mangrove and non-mangrove samples, respectively. CLC Genomics Workbench version 9.5.2 was used to assemble the filtered high-quality reads from the mangrove and non-mangrove data into scaffolds^29^. Genes were predicted from the assembled scaffolds using Prodigal-2.6.3, applied in the default parameter. The metagenomic sequences are submitted to NCBI under accession numbers SRR6811659 and SRR6821719 for mangrove and non-mangrove soil samples, respectively.

### Analysis of microbial taxonomy

Precise taxonomic categorization of high-throughput metagenomic sequencing data results, was utilized to examine the anticipated genes from both specimens using Kaiju^30^. The kaiju background database- NCBI BLAST nr + euk including fungi and microbial eukaryotes was applied for taxonomic classification. Greedy run-mode with default parameter was used to process assembled metagenome, the Kaiju web interface operates efficiently on an average computer.

### Functional metagenomic analysis

Functional analysis of the genes from mangrove and non-mangrove samples was performed using the COGNIZER 31 stand-alone framework. This framework enables the simultaneous provision of COG, KEGG, Pfam, GO, and SEED subsystem annotations to individual sequences constituting Metagenomic datasets. Using a novel ‘directed search’ step in COGNIZER helps significantly reduce the overall compute requirement typically associated with such functional analysis.

### Statistical data analysis

Conventional statistical techniques were employed to determine the mean, standard deviation, and standard error, with *p*-values less than 0.05 deemed significant for all tests. The correlation ranges between data from proportion or relative abundance was ascertained using the Spearman rank correlation coefficient. Species-wise regression coefficients for bacteria versus corresponding bacteriophage were calculated using the standard formula. Linear regression was estimated using Graphpad® software, which uses standard statistical computation methods https://www.graphpad.com/scientific-software/prism/. Subsequently, the Heatmap analysis was arranged using Clustvis®, a web tool for visualizing the clustering of multivariate data^31^. InteractiVenn is an online web-based tool for data analysis^32^.

### Ethical approval and consent to participate

The submitted manuscript is not submitted in any other journal. This manuscript doesn’t involve using any live animal or human data or tissue. The data were directly obtained from the Environmental sample and used for research.

### Consent for publication

The approval for submitting the manuscript was received from ICAR- Central Inland Fisheries Research Institute.

## Results

### Analyses of physical–chemical and chemical properties

Mangrove and non-mangrove soil samples were subjected to various analyses to understand the soil samples’ physicochemical properties and environmental conditions (Fig. 1B). It revealed that the soils from mangrove plantations were alkaline. In contrast, those from the paddy fields were acidic. The acidity might have developed due to the prolonged use of acid-forming fertilizers like urea and less use of organic manures. Paddy field soil’s nitrogen and phosphorus contents were also much higher than the mangrove soil (almost 2.5 times) for regular use of fertilizers in the cultivated area. Both soils were in a textural class of sandy loam, but in the mangrove soil, silt content was higher, adjacent to the river, while the paddy field soil contained more clay parts. The salinity of mangrove soil was significantly higher because of regular flushing by high salt-containing river waters. Organic carbon was in the low to medium range for both types of soil components. The river water had high salinity and total hardness values (5000 ppm CaCO_3_ eq.) as collected in the pre-monsoon season. The soil samples were collected from two sites of Sundarbans for analysis exhibits pH 8.5 (± 0.2) and 5.3 (± 0.5); specific conductivity (µS cm^−1^) 7120 (± 28) and 464 (± 117); organic carbon (%) 0.51 (± 0.03) and 0.42 (± 0.23); total nitrogen (%) 0.089 (± 0.008) and 0.124 (± 0.015); available nitrogen (mg 100 g^−1^ soil) 2.8 (± 28) and 6.58 (± 0.43); available phosphorus (mg 100 g^−1^ soil) 6.19 (± 2.11) and 17.25 (± 0.53) for mangrove and non-mangrove site (Fig. 1B).

### Sequencing, quality control and gene prediction

After removing the adapter and low-quality sequences from the raw sequence data, 40,877,196 (2 × 150 bp) and 34,413,814 (2 × 150 bp) high-quality reads were obtained for mangrove and non-mangrove samples. The good reads from both samples were put together to make scaffolds. A total of 1,585,765 and 1,517,859 scaffolds were received, with average scaffold sizes of 476 bp and 438 bp for mangrove and non-mangrove samples, respectively. Gene prediction from mangrove and non-mangrove metagenomes shows 261,910 and 223,837 genes with a minimum length of 500 bp.

### Microbial diversity of the mangrove and non-mangrove ecosystem

**Figure 2:**
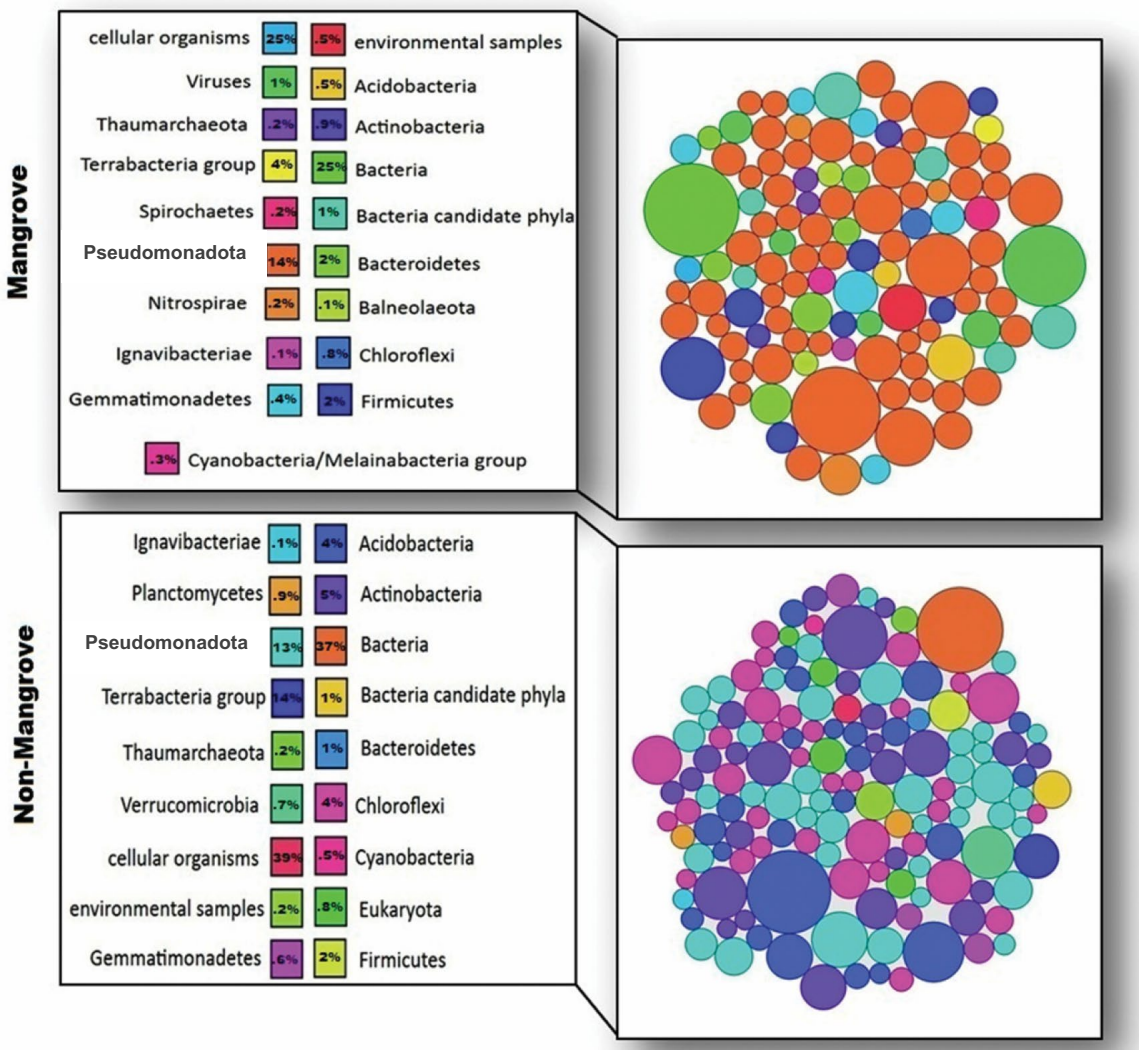
Important microbial population groups (Taxa) comprising at least 0.1% of classified reads between Sundarbans mangrove and non-mangrove environment. The bubble plot represents the number of reads assigned to each microbial population.

Taxonomic classification of microbes using a metagenome classifier (Kaiju-web interface) revealed that mangrove soil samples consist of 39% bacteria1% viruses and the remaining 60% of the reads could not be assigned to a taxa. Non-mangrove soil samples have 40% bacteria and 0.02% viruses. All other essential groups of microbial populations that occupied at least 0.1% of classified reads between the mangrove and non-mangrove environment were represented in Fig. 2.

### Methanogenic archaea species identified in Sundarbans

**Figure 3:**
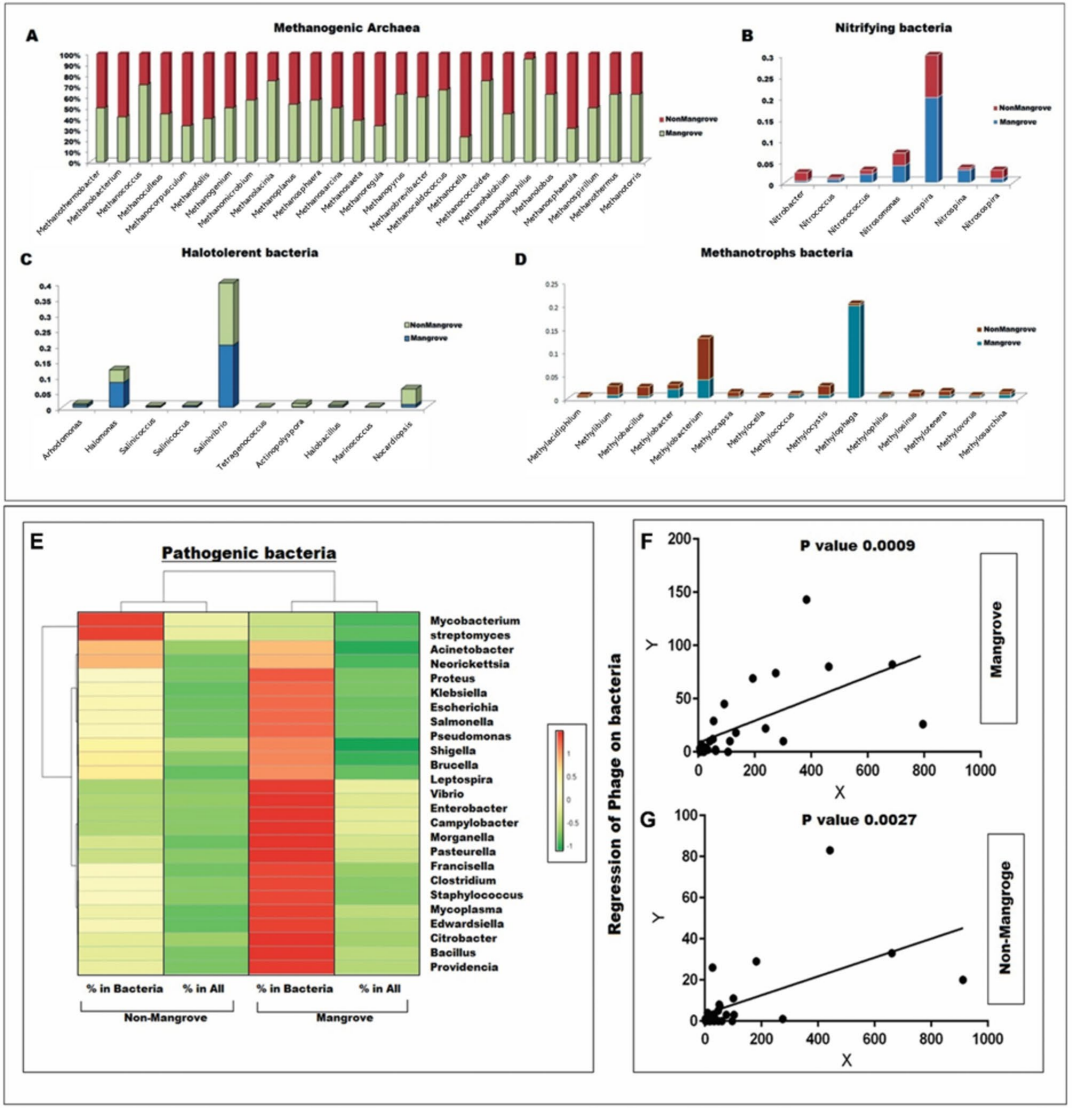
Relative abundance of the important genera of Methanogenic Archaea (**A**), Nitrifying Bacteria (**B**), Halotolerant Bacteria (**C**), and Methanotrophs Bacteria (**D**) found in mangrove and non-mangrove soil. Heat map representation of relative abundance of the important genus of pathogenic bacteria (**E**) Linear regression of Phage (%) on bacteria (%) found in mangrove (**F**) and non-mangrove (**G**) environment respectively.

A higher occurrence of genera such as *Methanolacinia, Methanohalophilus, Methanococcoides,* and *Methanothermus* was observed in the mangrove soil. A few genera like *Methanococcus, Methanocaldococcus, Methanosphaera, *etc*.* were present in both mangrove and non-mangrove soil samples (Fig. 3A). While the non-mangrove soils had a superior proportion of genera viz*., Methanomicrobium, Methanobacterium* and *Methanospirillum,* A comprehensive list of methanogenic species that are highly abundant in both mangrove and non-mangrove ecosystems is provided in Tables S1 and S8. A significant correlation (0.66, *p*-value 4 × 10^−8^) was found in the total count of archaea between mangrove and non-mangrove samples.

### Nitrifying bacteria identified in the Sundarbans

The nitrifying bacteria in the genera *Nitrococcus, Nitrosococcus, Nitrosomonas, Nitrospira, and Nitrospina* were abundant in the mangrove soil. In contrast, Nitrobacter and *Nitosospina* were only found in non-mangrove soil. Interestingly, the correlation abundance of genera between two places is highly significant numbers (0.97, *p*-value 2.9 × 10^−4^) (Fig. 3B). Nitrifying bacteria were also exceedingly abundant in mangroves as well as non-mangrove samples are described in Tables S2 and S9*;* the Correlation between the bacteria species within two ecosystems was found to be highly significant (0.44, *p*-value 4.5 × 10^−3^).

### Salt-tolerant/Halotolerant bacteria identified in mangrove soil

In mangrove soil, several salt-tolerant genera of bacteria like, *Arhodomonas, Halomonas, Halobacillus, Marinococcus, Salinicoccus, Salinivibrio, Staphylococcus and Tetragenococcus,* were reported (Fig. 3C). In non-mangrove soil; we found *Actinopolyspora* and *Nocardiopsis* are abundant genera of bacteria. A significant statistical correlation was observed between the mangrove and non-mangrove ecosystems for the relative abundance of percentage bacteria (0.98, *p*-value 8 × 10^−7^) and all microbes (0.95, *p*-value 2 × 10^−5^).

Species exceedingly abundant in mangrove soil are *A. haloalkaliphilus, H. anticariensis, Halomonas elongate,* etc. In non-mangrove soil, the predominant species were *Staphylococcus aureus, Actinopolyspora halophila, Actinopolyspora mzabensis,* etc. (Tables S3 and S10). Outputs also established a moderate statistically significant correlation for the percentage of bacteria (0.38 and *p*-value 1 × 10^−2^) between mangrove and non-mangrove soil.

### Methanotrophs bacterial species in Mangrove soil

The methanotrophs genera *Methylophaga, Methylophilus, Mythylobacter, Methylococcus, Methylovorus,* and *Methylosarcina* were highly abundant in mangrove soil. In contrast, *Methylocella, Methylobacillus, Methylosinus, Methylbium, Mehtylocaspa, Methylocystis, Methylacidiphilum* were the dominant group in non-mangrove soil (Fig. 3D). Dominant representative species available in mangrove soil were *M. tundripaludum, Methylobacter luteus* (Tables S4 and S11). The most abundant species available in non-mangrove soil were *Methylacidiphilum infernorum*, *Methylacidiphilum fumariolicum*, and other bacteria (Table S4). Statistically, a significant correlation was also found for a total number of counts (0.62, *p*-value 3 × 10^−4^), percentage of bacteria (0.58, *p*-value 8 × 10^−4^), percentage of all (0.58, *p*-value 8 × 10^−4^) microbes among the mangrove versus non-mangrove soil.

### Pathogenic bacteria identified in Sundarbans

Experimental data found bacterial genera, highly overflowing in Sundarbans, are *Salmonella, Acinetobacter, Aeromonas, Bacillus, Brucella, Campylobacter, Clostridium, Citrobacter, Edwardsiella, Enterobacter, Escherichia Francisella, Klebsiella, Leptospira, Mycoplasma, Pasteurella, Pseudomonas, Shigella, Staphylococcus, Vibrio,* etc*.* Only the genera *Mycobacterium* and *Actinomyces*were considerable numbers in the non-Sunderbans area (Fig. 3E). Correlation for the relative abundance of bacteria between the mangrove and non-mangrove samples was statistically significant (0.45, *p*-value 9 × 10^−3^).

Sundarbans mangrove and non-mangrove had a high abundance of critical pathogenic bacteria, details of which are given in Tables S5 and S12. Remarkably, there is a significant correlation between the relative abundance of bacterial genus versus the corresponding bacteriophage for mangrove (r-value 0.61) and non-mangrove soil (r-value 0.56). Consequently, the statistically significant regression abundance of phage was also found on bacterial genus for both mangrove (*p*-value 9 × 10^−4^) and non-mangrove (*p*-value 2.7 × 10^−3^) (Fig. 3F & G).

### Bioremediation bacteria identified in Sundarbans

**Figure 4:**
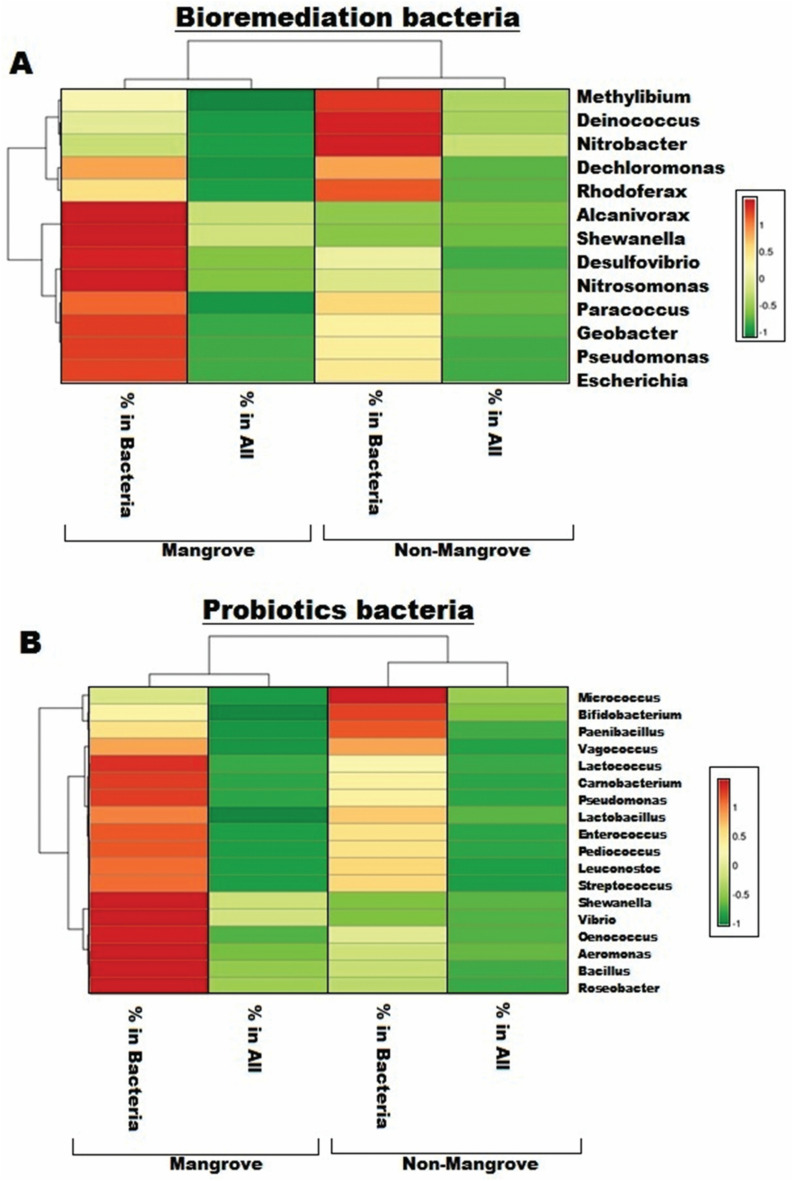
Heat map represents the relative abundance of the important genera of bioremediation bacteria (**A**) and Probiotics bacteria (**B**) found in mangrove and non-mangrove environments.

Analytical results observed that *Pseudomonas, Alcanivorax, Desulfovibrio, Escherichia, Geobacter, Nitrosomonas, Paracoccus, Shewanella* were abundant in mangrove soil, whereas in non-mangrove soil significant occurrences of *Dechloromonas, Deinococcus, Methylibium, Nitrobacter, Rhodoferax* (Fig. 4A). A higher statistically significant correlation was observed between two locations for bacteria percentage (0.75, *p*-value 3 × 10^−3^) and all (0.76, *p*-value 2 × 10^−3^). *Pseudomonas alcaligenes, P. mendocina,* and *Pseudomonas pseudoalcaligenes* were distributed in mangrove soil, whereas non-mangrove soil was dominated by the *Geobacter sulfurreducens, Geobacter metallireducens* (Tables S6 and S13). Therefore, a non-significant statistical correlation was found between the two soils’ relative abundance of bacteria species.

### Probiotics species identified in mangrove soils

Probiotics genera found in different numbers within Sundarbans were *Aeromonas, Bacillus, Carnobacterium, Enterococcus, Lactobacillus, Lactococcus, Leuconostoc, Oenococcus, Pediococcus, Pseudomonas, Roseobacter, Shewanella, Streptococcus* and *Vibrio*. We found that the dominant probiotics genera in non-mangroves soils were *Micrococcus, Bifidobacterium, Paenibacillus,* and *Vagococcus* (Fig. 4B). A Statistically significant correlation (0.76, *p*-value 3 × 10^−4^) was found for the relative abundance of bacteria genus between both the locations.

Probiotic species were highly abundant in Sundarbans and non-Sunderbans regions, as summarized in Tables S7 and S14). We found a significant correlation (0.44 and *p*-value 6 × 10^−4^) for the relative abundance of probiotics species among the total percentage between the mangroves versus non-mangrove samples.

### Functional metagenomic

**Figure 5:**
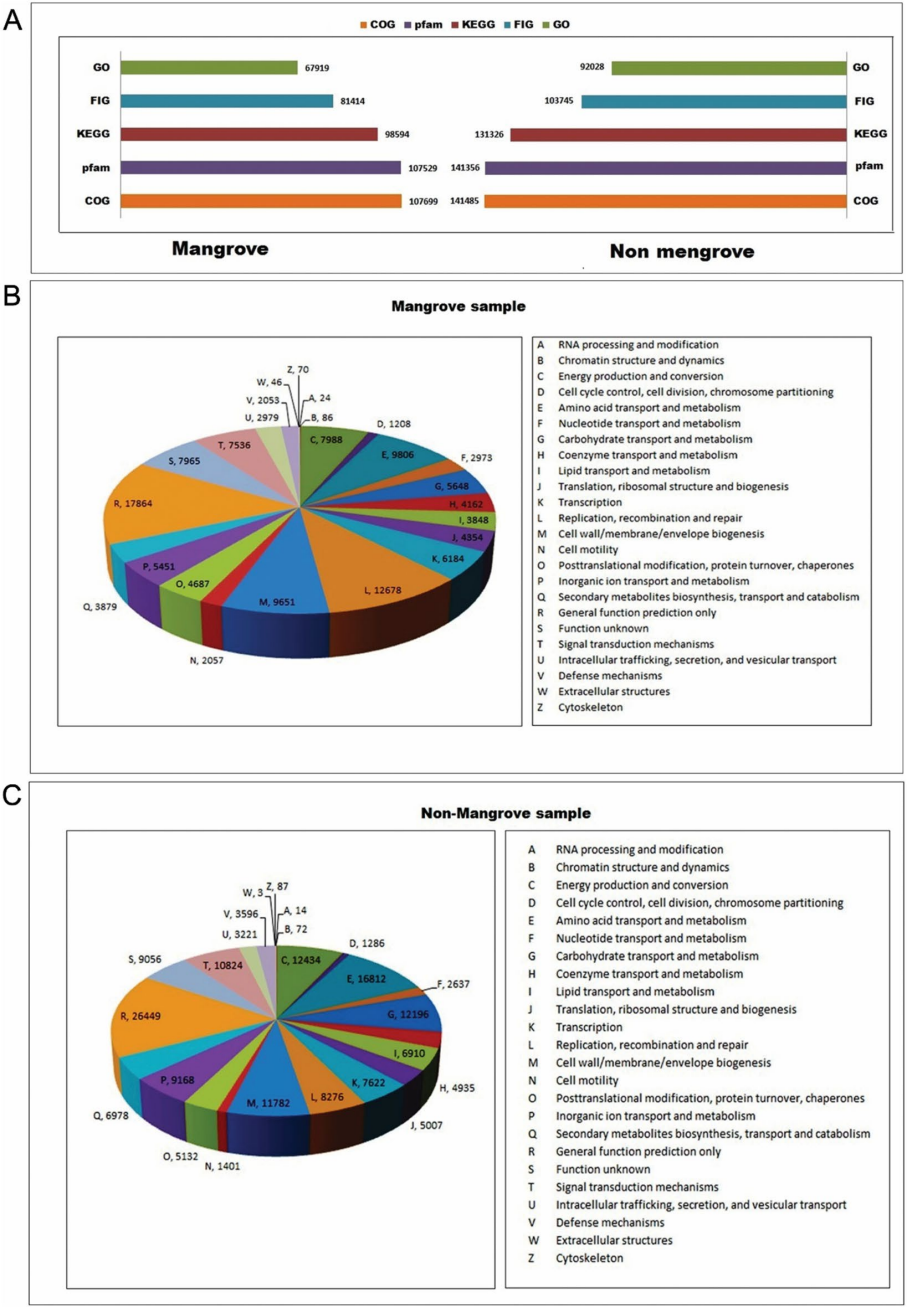
(**A**) Comparative functional metagenomic hit distribution between mangrove and non-mangrove area. (**B**) Metabolic pathway in the mangrove ecosystem. (**C**) Metabolic pathway in the non-mangrove ecosystem.

Functional metagenomics analysis was conducted to understand the assigned functional gene in mangroves and non-mangroves through COG, KEGG, Pfam, and GO. SEED subsystem annotations of individual sequences (Fig. 5A). The genes assigned to each pathway in mangrove soil were RNA processing and modification (24), chromatin structure and dynamics (86), nucleotide transport and metabolism (2973), replication recombination and repair (12,678), cell motility (2057) (Fig. 5B). Non-mangrove soil were enriched for more number pathways, *e.g.,* energy production and conversion (12,434), cell cycle control, cell division, chromosome partitioning (1286), amino acid transport and metabolism (16,812), carbohydrate transport and metabolism (12,196), transcription (7622), cell wall membrane, envelope, biogenesis (11,782), post-translational modification, protein turnover, chaperones (5132), inorganic ion transport and metabolism (9168), gene function prediction only (26,449), etc. (Fig. 5C).

When salt water enters the aquatic ecosystem, it may result in the fluctuation of microbial community structures, affecting sediment quality. Hence, assessing and understanding the microbial ecology of mangrove and non-mangrove samples is essential for developing management plans. The PCA biplot showed significant variability in bacterial abundance between the two sampling stations (Figure S1). The study found that mangrove samples were observed to be strongly influenced by pH, organic carbon, and specific conductivity.

### Inhabitation of microbes

**Figure 6:**
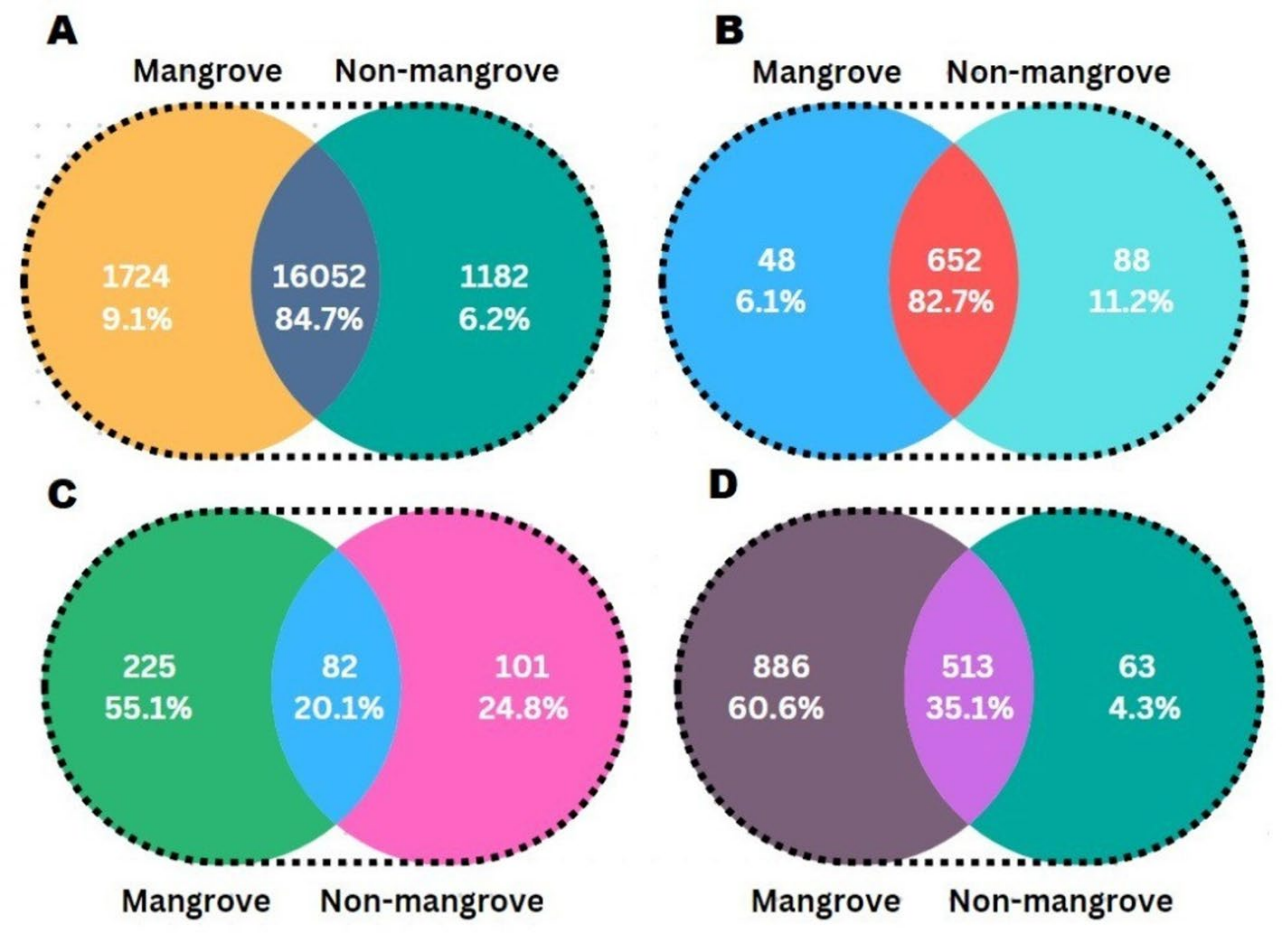
Venn diagram representing specific microorganisms in the Mangrove region and non-mangrove region. (**A**) Bacteria; (**B**) Fungi; (**C**) Virus and (**D**) Bacteriophage.

The current research design discovered 1970 bacterial species-specific for mangrove soil, 9029 particulars for non-mangrove soil, and 11,249 commons for both places (Fig. 6A). Veen diagram analysis explored 48 fungal species-specific for mangroves, 88 particulars for non-mangrove, and 652 were universal for both sites (Fig. 6B). Details are listed in parallel to those 225 viral species found specific to mangrove, 101 particulars for non-mangrove, and 82 frequent for both places (Fig. 6C). Many bacteriophages identified in mangrove, non-mangrove, and widely common to both soils were 886, 63, and 513, respectively (Fig. 6D).

## Discussion

Examining microbial diversity’s spatial arrangement in mangrove sediment remains insufficiently explored. A custom bioinformatic platform was developed, providing relative information on potential bioremediation, including probiotic species, nitrifying bacteria, and methanogenic and methanotrophic species from mangrove and non-mangrove ecosystems. The results underscore the importance of microbial communities in a mangrove ecosystem and demonstrate that physicochemical parameters of sediments are critical drivers of bacterial community structure in the sediments of the mangrove ecosystem. The microbial abundance and diversity exhibit significant variability in sediment quality parameters (Figure S1). The microbial abundance from mangrove samples was strongly influenced by pH, organic carbon and specific conductivity.

Our findings were explored through the robust taxonomical diversity of the microbiome in mangrove soil. The findings of Basak et al.^33^ can support our results; they also did a taxonomic study of 2746 species that belonged to 33 different phyla, revealing the dominance of *Pseudomonadota*, *Bacillota*, *Chloroflexota*, *Bacteroidota*, *Acidobacteriota*, *Nitrospirota,* and *Actinomycetota*, respectively. Remarkably, less than 5.0% of sequences belong to a poorly characterized group. However, these findings also need species-level information on microbial species. Our results also have similarities with those of Imchen et al.^34^ who recently reported microbial communities in mangrove sediment samples from four locations of an extensive mangrove system in Kerala, India. They found phylum *Pseudomonadota,* the major taxon among the forest soil samples. The researchers compared mangrove isolates from India, fragments from a Brazilian mangrove, and a North American tropical rainforest. Metagenomics analysis of the microbiome of the Brazilian mangrove sediments by Andreote et al. revealed the dominance of *Desulfobacterota* and *Gammaproteobacteria* in the samples, which confirms our results^35,36^.

Mangroves comprise a globally significant intertidal ecosystem with a high diversity of microorganisms, and members of the Mycobacterium and Actinomyces group, which are considered pathogenic bacteria, are dominant in the mangrove soil. The findings of de Magany et al.^37^ supported our results, who reported the role of zooplankton diversity in *Vibrio cholerae* population dynamics and the incidence of cholera in the Bangladesh Sundarbans (de Magny et al.^37^). Poharkar et al.^38^ collected samples from Goa, India, and ten mangrove ecosystems were studied by collecting samples and conducting total viable counts of pathogens like *E. coli*, *Listeria*, *Salmonella*, and *Vibrio* spp. The counts ranged from 1.25 to 3.9 × 103 cfu/mL, surpassing the relevant standards. These findings confirmed our results, which showed a high occurrence of pathogenic bacteria in mangrove soils.

We explored several salt-tolerant genera of bacteria for the first time, numerous of which were in the Sundarbans **(**Table S10). No detailed pictures of halotolerant bacteria from the Sundarbans were available, and our findings are unique in that context. A similar result was obtained by Rishad et al., who reported the biocontrol potential of halotolerant bacterial chitinase from the high-yielding novel *Bacillus pumilus* MCB-7^39^. Bibi et al.^15^ isolated 46 different rhizo and endophytic bacteria from the mangroves’ soil, roots, and leaves using various enzymatic media from the coastal area of Thuwal, Jeddah, Saudi Arabia. These bacterial strains could produce relevant enzymes (cellulase, protease, lipase, and amylase) and antibiotics with prospective industrial and medicinal uses. Our present research findings can be beneficial in guiding us in further investigation of unique enzymes from the Sundarbans ecosystems.

Detailed information on probiotic species from the Sundarbans still needs to be provided. The report described bacteriocin-producing lactic acid bacteria (*Lactococcus lactis* subsp. *lactis* KT2W2L) isolated from mangrove forests in southern Thailand as the only potential biocontrol agents^40,41^. We found *Lactococcus lactis* subsp. *lactis* CV56 and *Lactococcus lactis* subsp. *lactis* CNCM I-1631 in Sundarbans soil. We were the first to report several probiotic species identified from the Sundarbans Forest of West Bengal. These had many possible commercial applications (*e.g*., growth stimulators of aquatic species and human probiotics). We reported the following probiotic species in the Sundarbans, e.g., *V. mediterranei, V. fluvialis, Vibrio harveyi, B. pumilus, Bacillus licheniformis, L. curvatus, Lactobacillus helveticus, Lactobacillus buchneri, Lactobacillus gasseri, Lactobacillus rhamnosus*, etc.

The equilibrium between nitrification and denitrification processes in natural and managed ecosystems 44 influences global nitrous oxide (N_2_O) emissions. Nitrification consists of two stages: ammonia oxidation carried out by ammonia-oxidizing bacteria/archaea, and nitrite oxidation performed by nitrite-oxidizing bacteria. Microbes mainly drive this process and directly depend on nitrogen and the oxidation states of various elements (such as nitrate, iron, manganese, sulfur, acetate, etc.) in soils. Therefore, N_2_O emissions in mangrove ecosystems directly correlate with the ratio of nitrifiers to denitrifiers^42^. In Sundarbans mangrove soil, the genera of nitrifying bacteria *Nitrococcus, Nitrosococcus, Nitrosomonas, Nitrospira,* and *Nitrospina* were numerous compared to non-mangrove soil. In contrast, only *Nitrobacter* and *Nitosospina* were present in higher proportions. However, the abundance correlation between the two places was highly significant (*p*-value < 0.05). We have identified several nitrifying species that were highly plentiful in mangrove and non-mangrove soils as well (Table S9). Fan et al. (2015) reported different types of nitrifying bacteria and archaea in the coastal microbial mat at different tidal gradients on the North Sea beach of the Dutch barrier island Schiermonnikoog^43,44^. The reported bacteria include *Nitrosomonas marina* lineage, *Nitrosomonas communis* lineage, *Nitrosomonas europaea* lineage, and *Nitrosopira* clusters A, B, and C. As mentioned in the report, we found similar archaea in our present study, viz*., Nitrosopuilus, Nitrosotalea, Nitrososphaera,* and more.

In mangrove ecosystems, waterlogged soils create anaerobic conditions with low redox potential (approximately 200 mV), promoting methanogenesis. In this activity, CH_4_ is generated through the work of methanogenic archaebacteria that utilize acetate or CO_2_ as terminal electron acceptors^45,46^. We found that the Sundarban soil has a higher occurrence of the genera *Methanolacinia, Methanohalophilus, Methanococcoides,* and *Methanothermus* (Table S8). Bhattacharya et al. also studied the diversity and distribution of achaea in the mangrove sediments of the Sundarbans (Godkhali, Bonnie camp, and Dhulibhashani)^47^. The taxonomic examination uncovered the predominance of Euryarchaeota and Thaumarchaeota phyla. An increased presence of Halobacteriaceae was found in the subsurface samples studied by Godkhali and Bonnie. In contrast, samples from Dhulibhashani exhibited higher counts of Methanosarcinaceae and Methanobacteriaceae. Those reports needed species-level findings for comparison with our research findings. These findings also needed genus and species level information compared to our results, but the dominance of phyla *Euryarchaeota* was similar to our study. However, our results revealed quite diverse species of methanogenic archaea, which should have been reported earlier.

Our study found many methanotrophs, including the genus Methylophaga, Methylophilus, Mythylobacter, Methylococcus, Methylovorus, and Methylosarcina in mangrove soil. Shiau et al. found that in dwarf mangrove soils, Methylobacter and Methylosarcina played a pivotal role as active methanotrophs^43^. On the other hand, Methylomonas and Methylosarcina had a more significant presence in the tidal mangrove soils of Taiwan. Additionally, they observed variations in the methanotrophic community structure among coastal mangrove forests subjected to different tidal frequencies—however, species-level information needed to be updated. We were the first to provide a species-level comparison of methanotroph bacteria between mangrove soil and non-mangrove soil (Tables S4 and S11), which were very important to reducing the emission of greenhouse gases in the earth’s atmosphere.

The mangroves have been found to effectively capture trace metals in forms that are not bioavailable due to the quick formation of stable metal sulfides in anaerobic environments and their powerful bonding with organic complexes^48,49^. We discovered several bioremediation bacteria from mangrove and non-mangrove soil data, which actively cleaned our contaminated atmosphere. In mangrove soil, we found a high abundance of *P. alcaligenes, P. mendocina, Alcanivorax borkumensis, Desulfovibrio vulgaris, Desulfovibrio desulfuricans, Escherichia coli, P. denitrificans, S. putrefaciens,* etc. *P. alcaligenes* can degrade polycyclic aromatic hydrocarbons^50^, and *P. mendocina* can degrade toluene^51^. *Alcanivorax borkumensis* was reported for alkane-degradation in a marine environment, predominant in crude oil-containing seawater when nitrogen and phosphorus nutrients were supplemented^52^. We found several bio-remediation bacteria in our mangrove and non-mangrove soil, which were reported for different types of pollutant degradation.

A few pathways are enriched: RNA processing and modification, chromatin structure and dynamics, nucleotide transport and metabolism, replication recombination and repair, and cell motility in mangrove soil. Our work could be supported by the findings of Imchen et al., who reported that the top ten subsystems were membrane transport, virulence, disease and defence; RNA metabolism; cell wall and capsule; DNA metabolism; respiration; cofactors, vitamins, prosthetic groups, pigments; protein metabolism; amino acids and derivatives, carbohydrates found in Kerala mangrove, Brazilian mangrove, rain forest and Ocean sediments samples^53^.

We found several bacteria, viruses, bacteriophages, and fungi that were very specific to the Sunderban ecosystem and had not been described in detail earlier. Important bacterial species were *Actinobacillus rossii, Acetobacter peroxydans, Arcobacter halophilus, Bacillus gibsonii, Bacillus infantis, Bogoriella caseilytica, Campylobacter avium, Pelobacter massiliensis, Pseudomonas pavonaceae, Streptococcus downei, Vibrio cholerae* BJG-01, *Xanthomonas theicola* is mentioned among a few. We have listed details of bacteria species found in mangrove soils.

We have explored several species of fungi in mangrove soil are *Candida albicans, Candida maltose, Cryptococcus gattii CA1873, Aspergillus elongates, Exophiala aquamarina, Penicillium canescens, Diaporthe amygdale, Epichloe festucae, Myelochroa aurulenta, Neurospora crassa OR74A, Peltigera membranacea,* etc. In Sundarbans soil, we also found bioremediation fungal species *Phanerochaete chrysosporium* and *Pleurotus ostreatus* PC15. These fungi species are most commonly used for the degradation of polycyclic aromatic hydrocarbons (PAHs) due to their production of ligninolytic enzymes such as lignin peroxidase, manganese peroxidize, and laccase^54^.

In our experiment, several virus species were found in mangrove soil. Some of those were Simian mastadeno virus A, Porcine mastadeno virus A, African swine fever virus, Aves polyomavirus 1, Human polyomavirus 2, Salmon gill poxvirus, Roseolo virus, Canarypox virus, Swinepox virus, Volepox virus, Avian sarcoma virus, H1N1 subtype, Human herpesvirus 6, Macaca fuscata rhadino virus, Avian sarcoma virus 31, etc. and detail list are provided.

We also discovered several bacteriophages, an essential group of microorganisms, in mangrove soil. Examples of bacteriophage observed were *Acinetobacter* phage phiAB11, *Bacillus* phage SBP8a, *Campylobacter* phage PC5, *Cyanophage* S-RIM, *Erwinia* phage phiEa116, *Escherichia* phage CICC 80,001, *Enterobacter* phage phiEap-3, *Klebsiella* phage vB_KpnP_BIS33, *Lactococcus* phage bIL66M1, *Mycobacterium* phage *Madruga, Nostoc* phage N1, *Pseudoalteromonas* phage BS5, *Pectobacterium* phage PM1, *Shewanella* phage SppYZU05, *Synechococcus* phage SPGM99-19, *Shigella* phage pSf-1, *Vibrio* phage 1032, *Yersinia* phage YpP-G, etc.

## Conclusion

Through a combination of metagenomic profiling and measurements of sediment quality parameters, we have constructed a more comprehensive network analysis of microbial composition and their functional roles in mangrove sediments. The findings highlight that the salinity or pH of mangrove soil was significantly higher because of the regular flushing of saline river water. In contrast, non-mangrove soil was acidic, possibly due to the continuous use of some acid-forming fertilizers in paddy cultivation. Microorganisms exhibit different metabolic capabilities in mangrove and non-mangrove ecosystems. Mangrove soils in the Sundarbans hosted higher numbers of bacteria, archaea, fungi, and viruses than non-mangrove soil. We found that microorganisms residing in the sediment of mangroves were more proficient at depositing nitrogen, carbon dioxide, and methane, as well as stimulating organophosphate solubilization; together, these could benefit the growth of mangrove plants and reduce the emission of greenhouse gases. The diverse spectrum of the microbiome in the Sundarbans has provided deep insight into multiple species of methanotrophs, methanogenic, and probiotic bacteria, which would serve for individual study and simultaneously its application in aquaculture and carbon budget mitigations. The identified consortia of microbes from the Sundarbans will also be very useful in acid soil for pH correction. Moreover, further physiological and transcriptomics investigation is required to determine whether mangrove sediments might also affect microbial gene expression.

### Supplementary Information

Supplementary Information.

## Data Availability

The datasets generated and analysed during the current study are available from the corresponding author on reasonable request.
