# Supplementary material for: Comparative metagenomic analysis from Sundarbans ecosystems advances our understanding of microbial communities and their functional roles
Source: Sci Rep. 2024 Jul 13;14:16218. doi: 10.1038/s41598-024-67240-1 (PMC11246455; doi:10.1038/s41598-024-67240-1)
Supplement: Supplementary file 1 — Supplementary Information. [file 41598_2024_67240_MOESM1_ESM.doc]

**Supporting Information**

**Table S1.** Relative abundance methanogenic species in Sundarbans and non-mangrove area


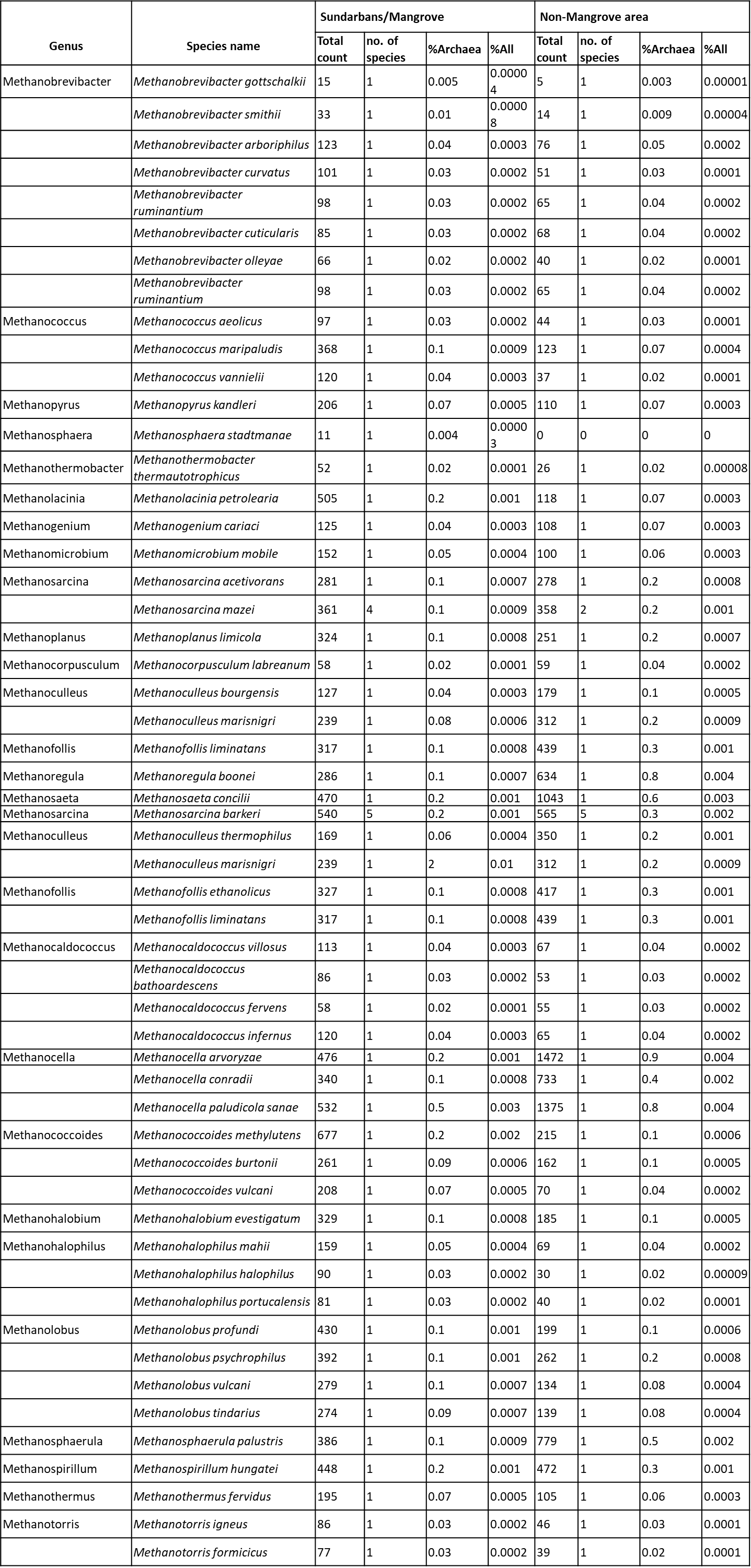


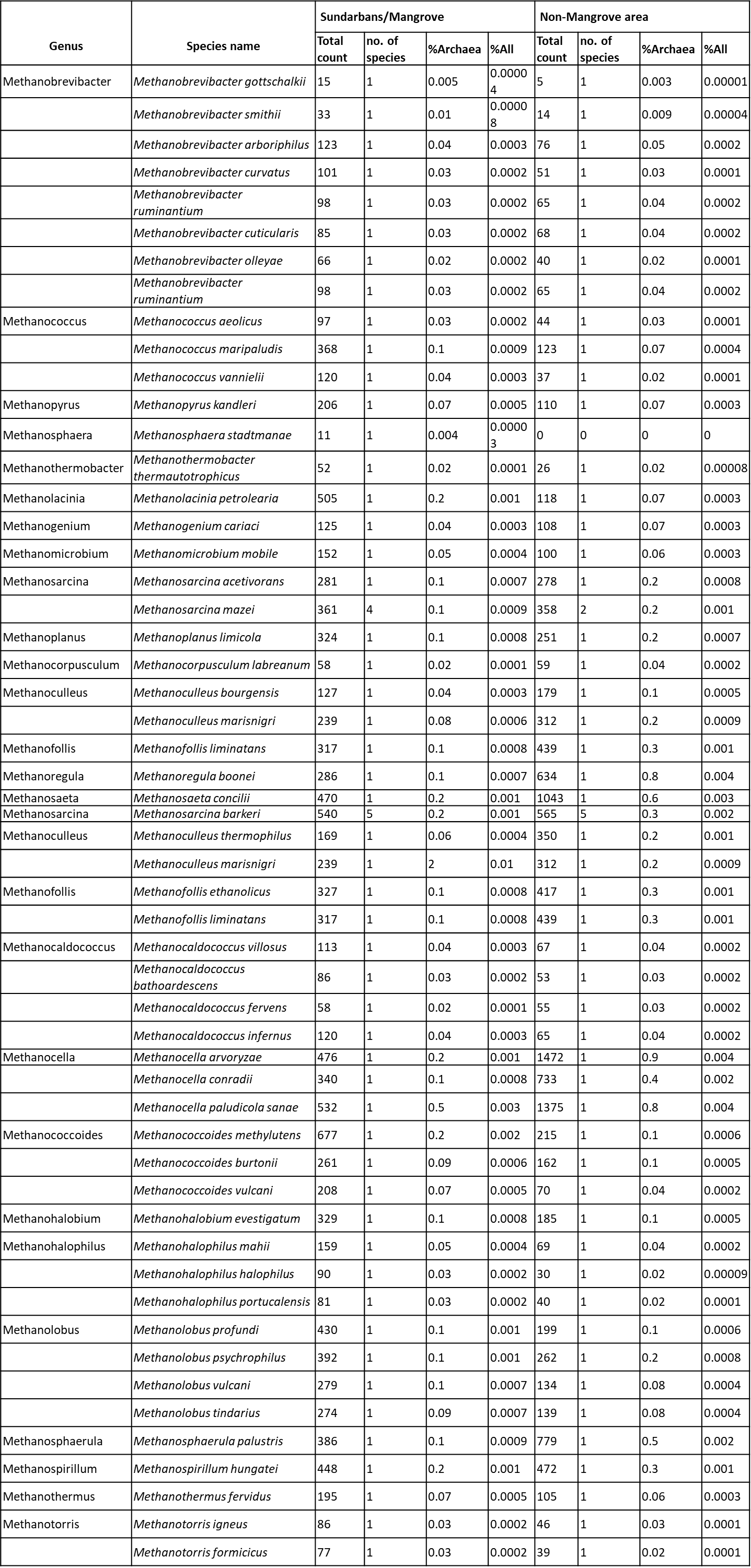


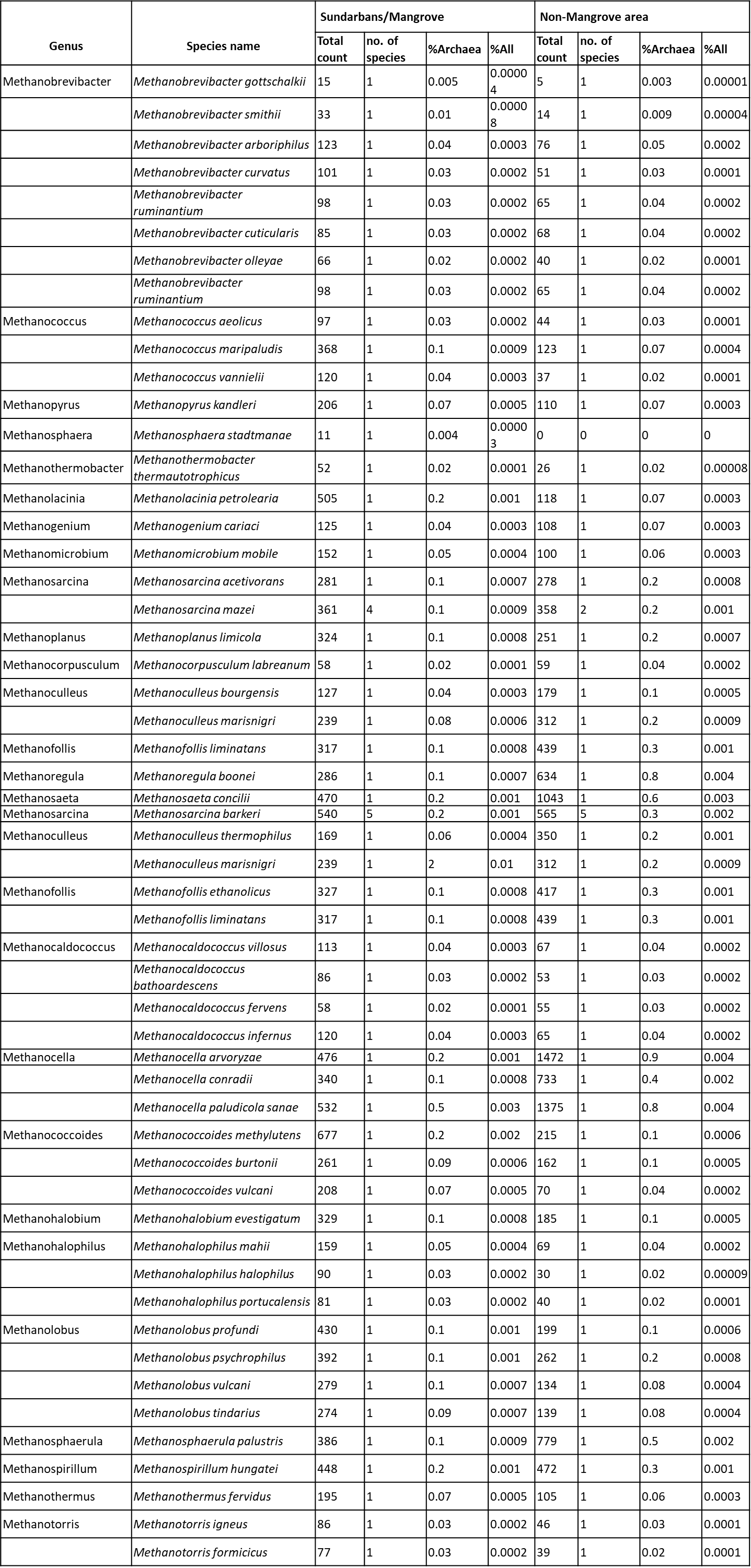


*Alphabets types in bold are more abundant in mangrove than in non-mangrove soil.

**Table S2.** Relative abundance of nitrifying bacteria in Mangrove and non-Mangrove area

|  |  | **Sundarbans/Mangrove** | | | **Non-Mangrove** | | |
| --- | --- | --- | --- | --- | --- | --- | --- |
| **Genus name** | **Species name** | **No. of species** | **% Bacteria** | **% All** | **No. of species** | **% Bacteria** | **% All** |
| *Nitrobacter* | *N. hamburgensis* | 1 | 0.004 | 0.001 | 1 | 0.01 | 0.005 |
|  | *N. vulgaris* | 1 | 0.002 | 0.0004 | 1 | 0.006 | 0.002 |
|  | *N. winogradskyi* | 1 | 0.003 | 0.0007 | 1 | 0.006 | 0.002 |
|  | *Nitrobacter sp. Nb-311A* | 1 | 0.003 | 0.0008 | 1 | 0.008 | 0.003 |
| *Nitrococcus* | *Nitrococcus mobilis* | **1** | **0.03** | **0.008** | **1** | **0.01** | **0.004** |
| *Nitrosococcus* | *Nitrosococcus oceani* | **3** | **0.02** | **0.005** | **3** | **0.008** | **0.003** |
|  | *Nitrosococcus halophilus* | **1** | **0.05** | **0.01** | **1** | **0.01** | **0.005** |
|  | *Nitrosococcus watsonii* | **1** | **0.01** | **0.003** | **1** | **0.005** | **0.002** |
| *Nitrosomonas* | *N. aestuarii* | **1** | **0.01** | **0.003** | **1** | **0.005** | **0.002** |
|  | *N. communis* | **1** | **0.009** | **0.002** | **1** | **0.007** | **0.003** |
|  | *N. europaea* | **1** | **0.005** | **0.001** | **1** | **0.003** | **0.001** |
|  | *N. eutropha* | **1** | **0.007** | **0.002** | **1** | **0.004** | **0.002** |
|  | *N. halophila* | **1** | **0.01** | **0.002** | **1** | **0.004** | **0.002** |
|  | *N. marina* | **1** | **0.02** | **0.004** | **1** | **0.004** | **0.002** |
|  | *N. nitrosa* | **1** | **0.007** | **0.002** | **1** | **0.004** | **0.001** |
|  | *N. oligotropha* | **1** | **0.003** | **0.0008** | **1** | **0.002** | **0.0007** |
|  | *N. ureae* | **1** | **0.007** | **0.002** | **1** | **0.004** | **0.001** |
|  | *N. mobilis* | **1** | **0.007** | **0.002** | **1** | **0.004** | **0.001** |
|  | *N. Cryotolerans* | **1** | **0.008** | **0.002** | **1** | **0.003** | **0.001** |
| *Nitrospira* | *N. moscoviensis* | **1** | **0.03** | **0.007** | **1** | **0.03** | **0.01** |
|  | *Nitrospira japonica* | **1** | **0.03** | **0.006** | **1** | **0.02** | **0.008** |
|  | *Nitrospira defluvii* | **1** | **0.007** | **0.002** | **1** | **0.006** | **0.002** |
|  | *Nitrospira sp. ST-bin5* | **1** | **0.02** | **0.005** | **1** | **0.01** | **0.005** |
|  | *Nitrospira sp. SG-bin2* | **1** | **0.02** | **0.004** | **1** | **0.007** | **0.002** |
| *Nitrospina* | *Nitrospina gracilis* | **1** | **0.1** | **0.02** | **1** | **0.009** | **0.003** |
|  | *Nitrospina sp. SCGC_AAA799_C22* | **1** | **0.02** | **0.004** | **1** | **0.003** | **0.001** |
|  | *Nitrospina sp. SCGC_AAA799_A02* | **1** | **0.007** | **0.002** | **1** | **0.001** | **0.0005** |
|  | *Nitrospina gracilis 3/211* | **1** | **0.003** | **0.0007** | **1** | **0.0006** | **0.0002** |
| *Nitrosospira* | *Nitrosospira multiformis* | 1 | 0.01 | 0.003 | 1 | 0.01 | 0.004 |
|  | *Nitrosospira briensis* | 1 | 0.006 | 0.001 | 1 | 0.007 | 0.002 |
|  | *Nitrosospira lacus* | 1 | 0.003 | 0.0008 | 1 | 0.003 | 0.0009 |
|  | *Nitrosospira sp. 56-18* | **1** | **0.008** | **0.002** | **1** | **0.006** | **0.002** |
|  | *Nitrosospira sp. Nsp14* | 1 | 0.006 | 0.001 | 1 | 0.006 | 0.002 |
|  | *Nitrosospira sp. Nl5* | 1 | 0.005 | 0.001 | 1 | 0.005 | 0.002 |
|  | *Nitrosospira sp. NpAV* | 1 | 0.005 | 0.001 | 1 | 0.003 | 0.001 |
|  | *Nitrosospira sp. Nsp11* | **1** | **0.002** | **0.0005** | **1** | **0.0009** | **0.0003** |
|  | *Nitrosospira sp. Nsp13* | 1 | 0.001 | 0.0004 | 1 | 0.002 | 0.0008 |
|  | *Nitrosospira sp. Nsp1* | 1 | 0.001 | 0.0003 | 1 | 0.001 | 0.0004 |
|  | *Nitrosospira sp. Nsp18* | 1 | **0.001** | **0.0002** | **1** | **0.0009** | **0.0003** |
|  | *Nitrosospira sp. 40KI* | 1 | **0.00002** | **0.000005** | **0** | **0** | **0** |

*Alphabets types in bold are most abundant in mangrove soil compared to non-mangrove soil.

**Table S3.** Salt tolerant bacteria identified in Mangrove Forest

|  |  | **Sundarbans/Mangrove** | | | **Non-Mangrove** | |  |
| --- | --- | --- | --- | --- | --- | --- | --- |
| **Sl. No.** | **Name of species** | **Total count** | **%Bacteria** | **%All** | **Total count** | **% Bacteria** | **%All** |
| 1 | *Alkalibacillus haloalkaliphilus* | **145** | **0.001** | **0.0004** | **110** | **0.0009** | **0.0003** |
| 2 | *Halomonas anticariensis* | **1152** | **0.01** | **0.003** | **577** | **0.005** | **0.002** |
| 3 | *Halomonas elongata* | **637** | **0.006** | **0.002** | **200** | **0.002** | **0.0006** |
| 4 | *Halomonas utahensis* | **1846** | **0.02** | **0.005** | **431** | **0.003** | **0.001** |
| 5 | *Halomonas muralis* | 756 | 0.008 | 0.002 | 395 | 0.003 | 0.001 |
| 6 | *Halomonas halmophila* | **2** | **0.00002** | **0.000005** | 0 | 0 | 0 |
| 7 | *Halomonas halodenitrificans* | **409** | **0.004** | **0.001** | **143** | **0.001** | **0.0004** |
| 8 | *Halomonas sp. Marseille-P2426* | **1312** | **0.01** | **0.003** | **123** | **0.001** | **0.0004** |
| 9 | *Halomonas ilicicola* | **733** | **0.007** | **0.002** | **372** | **0.003** | **0.001** |
| 10 | *Halomonas salina* | **787** | **0.008** | **0.002** | **269** | **0.002** | **0.0008** |
| 11 | *Halomonas korlensis* | **698** | **0.007** | **0.002** | **482** | **0.004** | **0.001** |
| 12 | *Marinococcus halotolerans* | **49** | **0.0005** | **0.0001** | **32** | **0.0002** | **0.00009** |
| 13 | *Salinicoccus albus* | **125** | **0.001** | **0.0003** | **79** | **0.0006** | **0.0002** |
| 14 | *Salinicoccus qingdaonensis* | **55** | **0.0005** | **0.0001** | **47** | **0.0004** | **0.0001** |
| 15 | *Salinicoccus luteus* | **44** | **0.0004** | **0.0001** | **34** | **0.0003** | **0.0001** |
| 16 | *Salinivibrio siamennsis* | **114** | **0.001** | **0.0003** | **22** | **0.0002** | **0.00006** |
| 17 | *Salinicoccus halodurans* | **57** | **0.0006** | **0.0001** | **57** | **0.0004** | **0.0002** |
| 18 | *Marinococcus luteus* | 35 | 0.0003 | 0.00009 | 74 | 0.0006 | 0.0002 |
| 19 | *Staphylococcus aureus* | 1665 | 0.02 | 0.004 | 59618 | 0.5 | 0.2 |
| 20 | *Actinopolyspora halophila* | 69 | 0.0007 | 0.0002 | 440 | 0.003 | 0.001 |
| 21 | *Salinicoccus alkaliphilus* | 103 | 0.001 | 0.0003 | 105 | 0.005 | 0.002 |
|  |  |  |  |  |  |  |  |
| **Sl. No.** | **Aerobic or facultatively anaerobic gram-negative moderately halophilic bacteria** | | | | | |  |
| 1 | *Salinivibrio costicola* | **1781** | **0.02** | **0.004** | **13** | **0.0001** | **0.00004** |
| 2 | *Arhodomonas aquaeolei* | 2948 | 0.03 | 0.007 | 1274 | 0.01 | 0.004 |
|  |  |  |  |  |  |  |  |
| **Sl. No.** | **Aerobic gram-positive moderately halophilic bacteria** | | |  |  |  |  |
| 1 | *Halobacillus halophilus* | 80 | 0.0008 | 0.0002 | 47 | 0.0004 | 0.0001 |
| 2 | *Halobacillus mangrovi* | **95** | **0.0009** | **0.0002** | **0** | **0** | **0** |
| 3 | *Halobacillus trueperi* | **39** | **0.0004** | **0.0001** | **18** | **0.0001** | **0.00005** |
| 4 | *Tetragenococcus muriaticus* | **62** | **0.0006** | **0.0002** | **57** | **0.0004** | **0.0002** |
| 5 | *Tetragenococcus halophilus* | **76** | **0.0008** | **0.0002** | **56** | **0.0004** | **0.0002** |
| 6 | *Marinococcus halophilus* | **68** | **0.0007** | **0.0002** | **67** | **0.0005** | **0.0002** |
| 7 | *Salinicoccus roseus* | **44** | **0.0004** | **0.0001** | **44** | **0.0003** | **0.0001** |
| 8 | *Halobacillus dabanensis* | 83 | 0.0008 | 0.0002 | 103 | 0.0008 | 0.0003 |
| 9 | *Halobacillus massiliensis* | 176 | 0.002 | 0.0004 | 199 | 0.002 | 0.0006 |
| 10 | *Halobacillus hunanensis* | 130 | 0.001 | 0.0003 | 151 | 0.001 | 0.0004 |
| 11 | *Halobacillus kuroshimensis* | 120 | 0.001 | 0.0003 | 202 | 0.002 | 0.0006 |
| 12 | *Tetragenococcus solitarius* | **46** | **0.0005** | **0.0001** | **49** | **0.0004** | **0.0001** |
| 13 | *Halobacillus salinus* | **110** | **0.001** | **0.0003** | **120** | **0.0009** | **0.0003** |
|  |  |  |  |  |  |  |  |
| **Sl. No.** | **Moderately halophilic actinomycete species** | | |  |  |  |  |
| 1 | *Actinopolyspora mortivallis* | 123 | 0.001 | 0.0003 | 535 | 0.004 | 0.002 |
| 2 | *Actinopolyspora mzabensis* | 74 | 0.0007 | 0.0002 | 246 | 0.002 | 0.0007 |
| 3 | *Nocardiopsis lucentensis* | 166 | 0.002 | 0.0004 | 483 | 0.004 | 0.001 |
| 4 | *Nocardiopsis halophila* | 33 | 0.0003 | 0.00008 | 128 | 0.001 | 0.0004 |
| 5 | *Nocardiopsis gilva* | 252 | 0.003 | 0.0006 | 899 | 0.007 | 0.003 |
| 6 | *Nocardiopsis salina* | 132 | 0.001 | 0.0003 | 543 | 0.004 | 0.002 |
| 7 | *Nocardiopsis halotolerans* | 68 | 0.0007 | 0.0002 | 438 | 0.003 | 0.001 |
| 8 | *Nocardiopsis potens* | 236 | 0.002 | 0.0006 | 1337 | 0.01 | 0.004 |

*Alphabets types in bold are most abundant in mangrove soil compared to non-mangrove soil

**Table S4.** Relative abundance of methanotrophs bacteria in Mangrove versus Non-Mangrove soils

|  |  | **Mangrove** | | | | **Non-Mangrove** | | | |
| --- | --- | --- | --- | --- | --- | --- | --- | --- | --- |
|  | **Species** | **total count** | **no. of species** | **% Bacteria** | **% All** | **total count** | **no. of species** | **% Bacteria** | **%All** |
| *Methylobacter* | *Methylobacter tundripaludum* | **2001** | **1** | **0.02** | **0.005** | **1178** | **1** | **0.009** | **0.003** |
|  | *Methylobacter luteus* | **1463** | **1** | **0.01** | **0.004** | **1004** | **1** | **0.008** | **0.003** |
|  | *Methylobacter marinus* | **300** | **1** | **0.003** | **0.0007** | **165** | **1** | **0.001** | **0.0005** |
|  | *Methylobacter whittenburyi* | **277** | **1** | **0.003** | **0.0007** | **154** | **1** | **0.001** | **0.0004** |
|  | *Methylobacter sp. BBA5.1* | **223** | **1** | **0.002** | **0.0005** | **144** | **1** | **0.001** | **0.0004** |
| *Methylococcus* | *Methylococcus capsulatus* | **2269** | **1** | **0.02** | **0.006** | **1231** | **1** | **0.01** | **0.004** |
|  | *Methylococcus capsulatus str. Bath* | **63** | **1** | **0.0006** | **0.0002** | **74** | **1** | **0.0006** | **0.0002** |
| *Methylophaga* | *Methylophaga thiooxydans* | **27259** | **1** | **0.3** | **0.07** | **290** | **1** | **0.002** | **0.0008** |
|  | *Methylophaga aminisulfidivorans* | **4944** | **1** | **0.05** | **0.01** | **113** | **1** | **0.0009** | **0.0003** |
|  | *Methylophaga lnarensis* | **4224** | **1** | **0.04** | **0.01** | **273** | **1** | **0.002** | **0.0008** |
|  | *Methylophaga sulfidovorans* | **3918** | **1** | **0.04** | **0.01** | **102** | **1** | **0.0008** | **0.0003** |
|  | *Methylophaga frappieri* | **3743** | **1** | **0.04** | **0.009** | **241** | **1** | **0.002** | **0.0007** |
|  | *Methylophaga nitratireducenticrescens* | **2709** | **1** | **0.03** | **0.007** | **213** | **1** | **0.002** | **0.0006** |
|  | *Methylophaga muralis* | **2634** | **1** | **0.03** | **0.006** | **215** | **1** | **0.002** | **0.0006** |
| *Methylophilus* | *Methylophilus methylotrophus* | **132** | **1** | **0.001** | **0.0003** | **89** | **1** | **0.0007** | **0.0003** |
|  | *Methylophilus sp. TWE2* | **118** | **1** | **0.001** | **0.0003** | **104** | **1** | **0.0008** | **0.0003** |
|  | *Methylophilus sp. 5* | 144 | 1 | 0.001 | 0.0004 | 148 | 1 | 0.001 | 0.0004 |
|  | *Methylophilus rhizosphaerae* | 191 | 1 | 0.002 | 0.0005 | 204 | 1 | 0.002 | 0.0006 |
| *Methylovorus* | *Methylovorus sp. MM1* | **763** | **1** | **0.008** | **0.002** | **492** | **1** | **0.004** | **0.001** |
|  | *Methylovorus glucosotrophus* | **81** | **1** | **0.0008** | **0.0002** | **78** | **1** | **0.0006** | **0.0002** |
|  | *Methylovorus sp. MP688* | 49 | 1 | 0.0005 | 0.0001 | 60 | 1 | 0.0005 | 0.0002 |
| *Methylosarcina* | *Methylos arcinafibrata* | **1795** | **1** | **0.02** | **0.004** | **1290** | **1** | **0.01** | **0.004** |
|  | *Methylos arcinalacus* | **1317** | **1** | **0.01** | **0.003** | **909** | **1** | **0.007** | **0.003** |
| *Methylacidiphilum* | *Methylacidiphilum infernorum* | 290 | 1 | 0.003 | 0.0007 | 523 | 1 | 0.004 | 0.002 |
|  | *Methylacidiphilum fumariolicum* | 102 | 1 | 0.001 | 0.0002 | 228 | 1 | 0.002 | 0.0007 |
|  | *Methylacidiphilum kamchatkense* | 87 | 1 | 0.0009 | 0.0002 | 162 | 1 | 0.001 | 0.0005 |
| *Methylibium* | *Methylibium sp. CF059* | 687 | 1 | 0.007 | 0.002 | 1771 | 1 | 0.01 | 0.005 |
|  | *Methylibium sp. NZG* | 495 | 1 | 0.005 | 0.001 | 1305 | 1 | 0.01 | 0.004 |
|  | *Methylibium petroleiphilum* | 149 | 1 | 0.001 | 0.0004 | 171 | 1 | 0.001 | 0.0005 |
| *Methylobacillus* | *Methylobacillus sp. MM2* | 1045 | 1 | 0.01 | 0.003 | 1232 | 1 | 0.01 | 0.004 |
|  | *Methylobacillus flagellatus* | 489 | 1 | 0.005 | 0.001 | 1085 | 1 | 0.008 | 0.003 |
|  | *Methylobacillus glycogenes* | 326 | 1 | 0.003 | 0.0008 | 3572 | 1 | 0.03 | 0.01 |
| *Methylobacterium* | *Methylobacteriu mnodulans* | 1033 | 1 | 0.01 | 0.003 | 2368 | 1 | 0.02 | 0.007 |
|  | *Methylobacterium aquaticum* | 534 | 1 | 0.005 | 0.001 | 1021 | 1 | 0.008 | 0.003 |
|  | *Methylobacterium extorquens* | 523 | 1 | 0.005 | 0.001 | 742 | 1 | 0.006 | 0.002 |
|  | *Methylobacterium populi* | 391 | 1 | 0.004 | 0.001 | 863 | 1 | 0.007 | 0.003 |
|  | *Methylobacterium mesophilicum* | 252 | 1 | 0.003 | 0.0006 | 737 | 1 | 0.006 | 0.002 |
| *Methylocapsa* | *Methylocapsa palsarum* | 557 | 1 | 0.006 | 0.001 | 1657 | 1 | 0.01 | 0.005 |
|  | *Methylocapsa acidiphila* | 513 | 1 | 0.005 | 0.001 | 1600 | 1 | 0.01 | 0.005 |
|  | *Methylocapsa aurea* | 471 | 1 | 0.005 | 0.001 | 1475 | 1 | 0.01 | 0.004 |
| *Methylocella* | *Methylocella silvestris* | 540 | 1 | 0.005 | 0.001 | 1742 | 1 | 0.01 | 0.005 |
| *Methylocystis* | *Methylocystis bryophila* | 673 | 1 | 0.007 | 0.002 | 1685 | 1 | 0.01 | 0.005 |
|  | *Methylocystis parvus* | 466 | 1 | 0.005 | 0.001 | 1423 | 1 | 0.01 | 0.004 |
|  | *Methylocystis rosea* | 172 | 1 | 0.002 | 0.0004 | 417 | 1 | 0.003 | 0.001 |
| *Methylosinus* | *Methylosinus sp. PW1* | 210 | 1 | 0.002 | 0.0005 | 546 | 1 | 0.004 | 0.002 |
|  | *Methylosinus sp. LW4* | 193 | 1 | 0.002 | 0.0005 | 393 | 1 | 0.003 | 0.001 |
|  | *Methylosinus sp. R-45379* | 89 | 1 | 0.0009 | 0.0002 | 176 | 1 | 0.001 | 0.0005 |
|  | *Methylosinus sp. LW3* | 63 | 1 | 0.0006 | 0.0002 | 263 | 1 | 0.002 | 0.0008 |
|  | *Methylosinus trichosporium* | 15 | 1 | 0.0001 | 0.00004 | 36 | 1 | 0.0003 | 0.0001 |
| *Methylotenera* | *Methylotenera versatilis* | 669 | 1 | 0.007 | 0.002 | 737 | 1 | 0.006 | 0.002 |
|  | *Methylotenera mobilis* | 441 | 1 | 0.004 | 0.001 | 454 | 1 | 0.004 | 0.001 |
|  | *Methylotenera sp. G11* | 403 | 1 | 0.004 | 0.001 | 1046 | 1 | 0.008 | 0.003 |
|  | *Methylotenera sp. L2L1* | 138 | 1 | 0.001 | 0.0003 | 147 | 1 | 0.001 | 0.0004 |

*Alphabets types in bold are most abundant in mangrove soil compared to non-mangrove soil.

**Table S5.** Relative abundance of pathogenic bacteria in Mangrove and non-Mangrove area

|  | **Sundarbans/Mangrove (M)** | | | **Non-Mangrove area (N)** | | | **Differences (M-N)** | | |
| --- | --- | --- | --- | --- | --- | --- | --- | --- | --- |
| **Genus/name** | **Total count** | **%Bacteria** | **%All** | **Total count** | **%Bacteria** | **%All** | **Total count** | **%Bacteria** | **%All** |
| *Pseudomonas aeruginosa* | **43** | **0.04** | **0.009** | **38** | **0.02** | **0.008** | **5** | **0.02** | **0.001** |
| *Vibrio cholerae* | **24** | **0.02** | **0.004** | **5** | **0.004** | **0.001** | **19** | **0.016** | **0.003** |
| *Clostridium botulinum* | **19** | **0.02** | **0.004** | **11** | **0.006** | **0.002** | **8** | **0.014** | **0.002** |
| *Mycoplasma capricolum* | **2** | **0.0006** | **0.0001** | **1** | **0.00002** | **9E-06** | **1** | **0.00058** | **0.000091** |
| *Leptospira interrogans* | **10** | **0.003** | **0.0007** | **5** | **0.0009** | **0.0003** | **5** | **0.0021** | **0.0004** |
| *Francisella noatunensis* | **3** | **0.002** | **0.0005** | **9** | **0.00007** | **0.00003** | **-6** | **0.00193** | **0.00047** |
| *Enterobacter cloacae* | **13** | **0.01** | **0.003** | **8** | **0.003** | **0.001** | **5** | **0.007** | **0.002** |
| *Klebsiella pneumoniae* | **25** | **0.01** | **0.003** | **26** | **0.006** | **0.002** | **-1** | **0.004** | **0.001** |
| *Edwardsiella tarda* | **3** | **0.002** | **0.0006** | **1** | **0.0006** | **0.0002** | **2** | **0.0014** | **0.0004** |
| *Citrobacte rfreundii* | **1** | **0.002** | **0.0005** | **1** | **0.0005** | **0.0002** | **0** | **0.0015** | **0.0003** |
| *Proteus mirabilis* | **2** | **0.002** | **0.0005** | **2** | **0.001** | **0.0004** | **0** | **0.001** | **0.0001** |
| *Morganella morganii* | **1** | **0.003** | **0.0008** | **1** | **0.0007** | **0.0003** | **0** | **0.0023** | **0.0005** |
| *Providencia stuartii* | **2** | **0.004** | **0.0009** | **2** | **0.001** | **0.0004** | **0** | **0.003** | **0.0005** |
| *Campylobacter fetus* | **2** | **0.008** | **0.002** | **1** | **0.001** | **0.0004** | **1** | **0.007** | **0.0016** |
| *Brucella abortus* | **2** | **0.002** | **0.0005** | **2** | **0.0003** | **0.0001** | **0** | **0.0017** | **0.0004** |
| *Salmonella enterica* | **87** | **0.02** | **0.006** | **48** | **0.02** | **0.006** | **39** | **0** | **0** |
| *Escherichia coli* | **175** | **0.05** | **0.01** | **90** | **0.03** | **0.01** | **85** | **0.02** | **0** |
| *Bacillus cereus* | **1** | **0.02** | **0.004** | **25** | **0.01** | **0.004** | **-24** | **0.01** | **0** |
| *Clostridium tetani* | **1** | **0.002** | **0.0004** | **2** | **0.0007** | **0.0003** | **-1** | **0.0013** | **0.0001** |
| *Pasteurella multocida* | **4** | **0.005** | **0.001** | **5** | **0.001** | **0.0004** | **-1** | **0.004** | **0.0006** |
| *Mycobacterium tuberculosis* | 21 | 0.01 | 0.003 | 37 | 0.04 | 0.01 | -16 | -0.03 | -0.007 |
| *Acinetobacter baumannii* | 44 | 0.02 | 0.004 | 29 | 0.1 | 0.05 | 15 | -0.08 | -0.046 |
| *Staphylococcus aureus* | 23 | 0.02 | 0.004 | 16 | 0.5 | 0.2 | 7 | -0.48 | -0.196 |
| *Shigella sonnei* | 1 | 0.003 | 0.0007 | 1 | 0.004 | 0.001 | 0 | -0.001 | -0.0003 |
| *Brucella suis* | 2 | 0.0004 | 0.0001 | 2 | 0.0005 | 0.0002 | 0 | -0.0001 | -0.0001 |
| *Brucella melitensis* | 4 | 0.0002 | 0.00006 | 3 | 0.0006 | 0.0002 | 1 | -0.0004 | -0.00014 |
| *Salmonella enterica subsp. enterica serovar Typhi* | 1 | 0.0005 | 0.0001 | 44 | 0.006 | 0.002 | -43 | -0.0055 | -0.0019 |

*Alphabets types in bold are most abundant in mangrove soil compared to non-mangrove soil

**Table S6.** Relative abundance of bioremediation bacteria in Mangrove and non-Mangrove area

| **Sl. No.** | **Name of species** | **Sundarbans/Mangrove (M)** | | | **Non-Mangrove (N)** | | | **Differences (M-N)** | | |
| --- | --- | --- | --- | --- | --- | --- | --- | --- | --- | --- |
|  |  | **Total count** | **%Bacteria** | **%All** | **Total count** | **%Bacteria** | **%All** | **Total**  **count** | **%Bacteria** | **%All** |
| 1 | *Pseudomonas alcaligenes* | **1082** | **0.01** | **0.003** | **710** | **0.006** | **0.002** | **372** | **0.004** | **0.001** |
| 2 | *Pseudomonas mendocina* | **9851** | **0.1** | **0.02** | **318** | **0.002** | **0.0009** | **9533** | **0.098** | **0.0191** |
| 3 | *Pseudomonas pseudoalcaligenes* | **551** | **0.005** | **0.001** | **362** | **0.003** | **0.001** | **189** | **0.002** | **0** |
| 4 | *Pseudomonas resinovorans* | **957** | **0.01** | **0.002** | **745** | **0.006** | **0.002** | **212** | **0.004** | **0** |
| 5 | *Pseudomonas veronii* | **123** | **0.001** | **0.0003** | **96** | **0.0007** | **0.0003** | **27** | **0.0003** | **0** |
| 6 | *Pseudomonas putida* | **1774** | **0.02** | **0.004** | **1233** | **0.01** | **0.004** | **541** | **0.01** | **0** |
| 7 | *Alcanivorax borkumensis* | **91** | **0.0009** | **0.0002** | **4** | **0.00003** | **0.00001** | **87** | **0.00087** | **0.0002** |
| 8 | *Desulfovibrio vulgaris* | **1176** | **0.01** | **0.003** | **770** | **0.006** | **0.002** | **406** | **0.004** | **0.001** |
| 9 | *Desulfovibrio desulfuricans* | **1704** | **0.02** | **0.004** | **848** | **0.007** | **0.002** | **856** | **0.013** | **0.002** |
| 10 | *Escherichia coli* | **5103** | **0.05** | **0.01** | **4240** | **0.03** | **0.01** | **863** | **0.02** | **0** |
| 11 | *Paracoccus denitrificans* | **292** | **0.003** | **0.0007** | **178** | **0.001** | **0.0005** | **114** | **0.002** | **0.0002** |
| 12 | *Shewanella putrefaciens* | **107** | **0.001** | **0.0003** | **42** | **0.0003** | **0.0001** | **65** | **0.0007** | **0.0002** |
| 13 | *Geobacter sulfurreducens* | 1428 | 0.01 | 0.003 | 1270 | 0.01 | 0.004 | 158 | 0 | -0.001 |
| 14 | *Geobacter metallireducens* | 2333 | 0.02 | 0.006 | 2452 | 0.02 | 0.007 | -119 | 0 | -0.001 |
| 15 | *Methylibium petroleiphilum* | 149 | 0.001 | 0.0004 | 171 | 0.001 | 0.0005 | -22 | 0 | -1E-04 |
| 16 | *Nitrosomonas europaea* | 505 | 0.005 | 0.001 | 351 | 0.003 | 0.001 | 154 | 0.002 | 0 |
| 17 | *Nitrobacter hamburgensis* | 408 | 0.004 | 0.001 | 1635 | 0.01 | 0.005 | -1227 | -0.006 | -0.004 |
| 18 | *Rhodoferax ferrireducens* | 692 | 0.007 | 0.002 | 1383 | 0.01 | 0.004 | -691 | -0.003 | -0.002 |
| 19 | *Pseudomonas stutzeri* | 2588 | 0.03 | 0.006 | 6506 | 0.05 | 0.02 | -3918 | -0.02 | -0.014 |
| 20 | *Dechloromonas aromatica* | 562 | 0.006 | 0.001 | 650 | 0.005 | 0.002 | -88 | 0.001 | -0.001 |
| 21 | *Deinococcus radiodurans* | 201 | 0.002 | 0.0005 | 442 | 0.003 | 0.001 | -241 | -0.001 | -5E-04 |

*Alphabets types in bold are most abundant in mangrove soil compared to non-mangrove soil.

**Table S7.** Relative abundance of probiotics bacteria in Mangrove and non-Mangrove area

| **Bacterial species** | **Mangrove (M)** | | | **Non- Mangrove (N)** | | | **Difference (M-N)** | | |
| --- | --- | --- | --- | --- | --- | --- | --- | --- | --- |
|  | **Total count** | **% Bacteria** | **% All** | **Total count** | **% Bacteria** | **% All** | **Total count** | **% Bacteria** | **% All** |
| *Vibrio mediterranei* | **24** | **0.0002** | **0.00006** | **10** | **0.00008** | **0.00003** | **14** | **0.00012** | **0.00003** |
| *Vibrio fluvialis* | **884** | **0.009** | **0.002** | **39** | **0.0003** | **0.0001** | **845** | **0.0087** | **0.0019** |
| *Vibrio harveyi* | **325** | **0.1** | **0.03** | **57** | **0.0004** | **0.0002** | **268** | **0.0996** | **0.0298** |
| *Vibrio alginolyticus* | **910** | **0.1** | **0.03** | **123** | **0.01** | **0.004** | **787** | **0.09** | **0.026** |
| *Bacillus Clausii* | **95** | **0.0009** | **0.0002** | **86** | **0.0007** | **0.0002** | **9** | **0.0002** | **0** |
| *Bacillus subtilis* | **9234** | **0.09** | **0.02** | **185** | **0.001** | **0.0005** | **9049** | **0.089** | **0.0195** |
| *Bacillus cereus* | **1516** | **0.02** | **0.004** | **1256** | **0.01** | **0.004** | **260** | **0.01** | **0** |
| *Bacillus mycoides* | **151** | **0.002** | **0.0004** | **88** | **0.0007** | **0.0003** | **63** | **0.0013** | **0.0001** |
| *Bacillus pumilus* | **299** | **0.003** | **0.0007** | **164** | **0.001** | **0.0005** | **135** | **0.002** | **0.0002** |
| *Bacillus Licheniformis* | **13977** | **0.1** | **0.03** | **142** | **0.001** | **0.0004** | **13835** | **0.099** | **0.0296** |
| *Lactobacillus Curvatus* | **59** | **0.0006** | **0.0001** | **35** | **0.0003** | **0.0001** | **24** | **0.0003** | **0** |
| *Lactobacillus helveticus* | **31** | **0.0003** | **0.00008** | **29** | **0.0002** | **0.00008** | **2** | **0.0001** | **0** |
| *Lactobacillus buchneri* | **31** | **0.0003** | **0.00008** | **13** | **0.0001** | **0.00004** | **18** | **0.0002** | **0.00004** |
| *Lactobacillus Gasseri* | **44** | **0.0004** | **0.0001** | **22** | **0.0002** | **0.00006** | **22** | **0.0002** | **0.00004** |
| *Lactobacillus rhamnosus* | **84** | **0.0008** | **0.0002** | **57** | **0.0004** | **0.0002** | **27** | **0.0004** | **0** |
| *Lactobacillus plantarum* | **140** | **0.001** | **0.0003** | **117** | **0.0009** | **0.0003** | **23** | **0.0001** | **0** |
| *Bifidobacterium bifidum* | **49** | **0.02** | **0.006** | **131** | **0.001** | **0.0004** | **-82** | **0.019** | **0.0056** |
| *Bifidobacterium longum* | **105** | **0.02** | **0.006** | **152** | **0.001** | **0.0004** | **-47** | **0.019** | **0.0056** |
| *Micrococcus luteus* | **83** | **0.003** | **0.0009** | **173** | **0.001** | **0.0005** | **-90** | **0.002** | **0.0004** |
| *Roseobacter litoralis Och 149* | **7** | **0.00007** | **0.00002** | **8** | **0.00006** | **0.00002** | **-1** | **1E-05** | **0** |
| *Lactobacillus delbrueckii* | **63** | **0.0006** | **0.0002** | **44** | **0.0003** | **0.0001** | **19** | **0.0003** | **0.0001** |
| *Lactobacillus salivarius* | **155** | **0.002** | **0.0004** | **90** | **0.0007** | **0.0003** | **65** | **0.0013** | **0.0001** |
| *Lactobacillus johnsonii* | **26** | **0.0003** | **0.00006** | **15** | **0.0001** | **0.00004** | **11** | **0.0002** | **0.00002** |
| *Lactobacillus reuteri* | **129** | **0.001** | **0.0003** | **83** | **0.0006** | **0.0002** | **46** | **0.0004** | **0.0001** |
| *Bifidobacterium adolescentis* | **131** | **0.02** | **0.006** | **110** | **0.0009** | **0.0003** | **21** | **0.0191** | **0.0057** |
| *Shewanella colwelliana* | **489** | **0.005** | **0.001** | **90** | **0.0007** | **0.0003** | **399** | **0.0043** | **0.0007** |
| *Shewanella putrefaciens* | **107** | **0.001** | **0.0003** | **42** | **0.0003** | **0.0001** | **65** | **0.0007** | **0.0002** |
| *Pediococcus pentosaceus* | **46** | **0.0005** | **0.0001** | **26** | **0.0002** | **0.00008** | **20** | **0.0003** | **0.00002** |
| *Pediococcus acidilactici* | **72** | **0.004** | **0.0009** | **59** | **0.0005** | **0.0002** | **13** | **0.0035** | **0.0007** |
| *Enterococcus durans* | **26950** | **0.3** | **0.07** | **178** | **0.03** | **0.01** | **26772** | **0.27** | **0.06** |
| *Streptococcus thermophilus* | **36** | **0.05** | **0.01** | **33** | **0.0003** | **0.0001** | **3** | **0.0497** | **0.0099** |
| *Leuconostoc mesenteroides* | **120** | **0.005** | **0.001** | **74** | **0.0006** | **0.0002** | **46** | **0.0044** | **0.0008** |
| *Aeromonas hydrophila* | **589** | **0.006** | **0.001** | **1** | **0.000008** | **0.000003** | **588** | **0.005992** | **0.000997** |
| *Paenibacillus polymyxa* | **471** | **0.005** | **0.001** | **296** | **0.002** | **0.0009** | **175** | **0.003** | **0.0001** |
| *Lactococcus lactis* | **389** | **0.3** | **0.07** | **251** | **0.002** | **0.02** | **138** | **0.298** | **0.05** |
| *Carnobacterium divergens* | **104** | **0.001** | **0.0003** | **87** | **0.0007** | **0.0003** | **17** | **0.0003** | **0** |
| *Carnobacteriumm altaromaticum* | **172** | **0.009** | **0.002** | **94** | **0.0007** | **0.0003** | **78** | **0.0083** | **0.0017** |
| *Roseobacter litoralis* | **421** | **0.004** | **0.001** | **8** | **0.00006** | **0.00002** | **413** | **0.00394** | **0.00098** |
| *Roseobacter denitrificans* | **305** | **0.03** | **0.008** | **91** | **0.0007** | **0.0003** | **214** | **0.0293** | **0.0077** |
| *Roseobacter denitrificans Och 114* | **9** | **0.00009** | **0.00002** | **7** | **0.00005** | **0.00002** | **2** | **0.00004** | **0** |
| *Vagococcus teuberi* | **23** | **0.0002** | **0.00006** | **18** | **0.0001** | **0.00005** | **5** | **0.0001** | **0.00001** |
| *Oenococcus alcoholitolerans* | **40** | **0.0004** | **0.0001** | **38** | **0.0003** | **0.0001** | **2** | **0.0001** | **0** |
| *Lactobacillus crispatus* | **108** | **0.001** | **0.0003** | **117** | **0.0009** | **0.0003** | **-9** | **0.0001** | **0** |
| *Bacillus circulans* | **96** | **0.001** | **0.0002** | **101** | **0.0008** | **0.0003** | **-5** | **0.0002** | **-0.0001** |
| *Lactobacillus casei* | 30 | 0.002 | 0.0004 | 29 | 0.002 | 0.0006 | 1 | 0 | -0.0002 |
| *Lactobacillus paracasei* | 56 | 0.0006 | 0.0001 | 41 | 0.002 | 0.0006 | 15 | -0.0014 | -0.0005 |
| *Lactobacillus acidophilus* | 10 | 0.0001 | 0.00002 | 13 | 0.0001 | 0.00004 | -3 | 0 | -0.00002 |
| *Bacillus megaterium* | 213 | 0.002 | 0.0005 | 193 | 0.002 | 0.0006 | 20 | 0 | -1.000E-04 |
| *Bifidobacterium animalis* | 44 | 0.0004 | 0.0001 | 113 | 0.0009 | 0.0003 | -69 | -0.0005 | -0.0002 |
| *Bifidobacterium breve* | 58 | 0.0006 | 0.0001 | 108 | 0.0008 | 0.0003 | -50 | -0.0002 | -0.0002 |
| *Pseudomonas fluorescens* | 2122 | 0.02 | 0.005 | 1992 | 0.02 | 0.006 | 130 | 0 | -0.001 |
| *Enterococcus faecium* | 306 | 0.003 | 0.0007 | 323 | 0.03 | 0.01 | -17 | -0.027 | -0.0093 |
| *Oenococcus oeni* | 110 | 0.001 | 0.0003 | 88 | 0.01 | 0.005 | 22 | -0.009 | -0.0047 |
| *Oenococcus kitaharae* | 35 | 0.0003 | 0.00009 | 46 | 0.0004 | 0.0001 | -11 | -0.0001 | -0.00001 |
| *Bacillus coagulans* | 346 | 0.003 | 0.0008 | 375 | 0.003 | 0.001 | -29 | 0 | -0.0002 |
| *Lactobacillus brevis* | 112 | 0.001 | 0.0003 | 190 | 0.001 | 0.0006 | -78 | 0 | -0.0003 |
| *Lactobacillus fermentum* | 104 | 0.001 | 0.0003 | 103 | 0.07 | 0.03 | 1 | -0.069 | -0.0297 |

*Alphabets types in bold are most abundant in mangrove soil compared to non-mangrove soil.

**Table S8. Relative abudance methanogenic species in Sundarbans and non-mangrove area**

| **Genus** | **Species name** | **Sundarbans/Mangrove** | | | | **Non-Mangrove area** | | | |
| --- | --- | --- | --- | --- | --- | --- | --- | --- | --- |
|  |  | **Total count** | **no. of species** | **%Archaea** | **%All** | **Total count** | **no. of species** | **%Archaea** | **%All** |
| *Methanobrevibacter* | *Methanobrevibactergottschalkii* | **15** | **1** | **0.005** | **0.00004** | **5** | **1** | **0.003** | **0.00001** |
|  | *Methanobrevibactersmithii* | **33** | **1** | **0.01** | **0.00008** | **14** | **1** | **0.009** | **0.00004** |
|  | *Methanobrevibacterarboriphilus* | 123 | 1 | 0.04 | 0.0003 | 76 | 1 | 0.05 | 0.0002 |
|  | *Methanobrevibactercurvatus* | **101** | **1** | **0.03** | **0.0002** | **51** | **1** | **0.03** | **0.0001** |
|  | *Methanobrevibacterruminantium* | 98 | 1 | 0.03 | 0.0002 | 65 | 1 | 0.04 | 0.0002 |
|  | *Methanobrevibactercuticularis* | 85 | 1 | 0.03 | 0.0002 | 68 | 1 | 0.04 | 0.0002 |
|  | *Methanobrevibacterolleyae* | **66** | **1** | **0.02** | **0.0002** | **40** | **1** | **0.02** | **0.0001** |
|  | *Methanobrevibacterruminantium* | 98 | 1 | 0.03 | 0.0002 | 65 | 1 | 0.04 | 0.0002 |
| *Methanococcus* | *Methanococcusaeolicus* | **97** | **1** | **0.03** | **0.0002** | **44** | **1** | **0.03** | **0.0001** |
|  | *Methanococcusmaripaludis* | **368** | **1** | **0.1** | **0.0009** | **123** | **1** | **0.07** | **0.0004** |
|  | *Methanococcusvannielii* | **120** | **1** | **0.04** | **0.0003** | **37** | **1** | **0.02** | **0.0001** |
| *Methanopyrus* | *Methanopyruskandleri* | **206** | **1** | **0.07** | **0.0005** | **110** | **1** | **0.07** | **0.0003** |
| *Methanosphaera* | *Methanosphaerastadtmanae* | **11** | **1** | **0.004** | **0.00003** | **0** | **0** | **0** | **0** |
| *Methanothermobacter* | *Methanothermobacterthermautotrophicus* | **52** | **1** | **0.02** | **0.0001** | **26** | **1** | **0.02** | **0.00008** |
| *Methanolacinia* | *Methanolaciniapetrolearia* | **505** | **1** | **0.2** | **0.001** | **118** | **1** | **0.07** | **0.0003** |
| *Methanogenium* | *Methanogeniumcariaci* | 125 | 1 | 0.04 | 0.0003 | 108 | 1 | 0.07 | 0.0003 |
| *Methanomicrobium* | *Methanomicrobium mobile* | 152 | 1 | 0.05 | 0.0004 | 100 | 1 | 0.06 | 0.0003 |
| *Methanosarcina* | *Methanosarcinaacetivorans* | 281 | 1 | 0.1 | 0.0007 | 278 | 1 | 0.2 | 0.0008 |
|  | *Methanosarcinamazei* | 361 | 4 | 0.1 | 0.0009 | 358 | 2 | 0.2 | 0.001 |
| *Methanoplanus* | *Methanoplanuslimicola* | 324 | 1 | 0.1 | 0.0008 | 251 | 1 | 0.2 | 0.0007 |
| *Methanocorpusculum* | *Methanocorpusculumlabreanum* | 58 | 1 | 0.02 | 0.0001 | 59 | 1 | 0.04 | 0.0002 |
| *Methanoculleus* | *Methanoculleusbourgensis* | 127 | 1 | 0.04 | 0.0003 | 179 | 1 | 0.1 | 0.0005 |
|  | *Methanoculleusmarisnigri* | 239 | 1 | 0.08 | 0.0006 | 312 | 1 | 0.2 | 0.0009 |
| *Methanofollis* | *Methanofollisliminatans* | 317 | 1 | 0.1 | 0.0008 | 439 | 1 | 0.3 | 0.001 |
| *Methanoregula* | *Methanoregulaboonei* | 286 | 1 | 0.1 | 0.0007 | 634 | 1 | 0.8 | 0.004 |
| *Methanosaeta* | *Methanosaetaconcilii* | 470 | 1 | 0.2 | 0.001 | 1043 | 1 | 0.6 | 0.003 |
| *Methanosarcina* | *Methanosarcinabarkeri* | 540 | 5 | 0.2 | 0.001 | 565 | 5 | 0.3 | 0.002 |
| *Methanoculleus* | *Methanoculleus thermophilus* | 169 | 1 | 0.06 | 0.0004 | 350 | 1 | 0.2 | 0.001 |
|  | *Methanoculleusmarisnigri* | 239 | 1 | 2 | 0.01 | 312 | 1 | 0.2 | 0.0009 |
| *Methanofollis* | *Methanofollisethanolicus* | 327 | 1 | 0.1 | 0.0008 | 417 | 1 | 0.3 | 0.001 |
|  | *Methanofollisliminatans* | 317 | 1 | 0.1 | 0.0008 | 439 | 1 | 0.3 | 0.001 |
| *Methanocaldococcus* | *Methanocaldococcusvillosus* | **113** | **1** | **0.04** | **0.0003** | **67** | **1** | **0.04** | **0.0002** |
|  | *Methanocaldococcusbathoardescens* | 86 | 1 | 0.03 | 0.0002 | 53 | 1 | 0.03 | 0.0002 |
|  | *Methanocaldococcusfervens* | 58 | 1 | 0.02 | 0.0001 | 55 | 1 | 0.03 | 0.0002 |
|  | *Methanocaldococcusinfernus* | **120** | **1** | **0.04** | **0.0003** | **65** | **1** | **0.04** | **0.0002** |
| *Methanocella* | *Methanocellaarvoryzae* | 476 | 1 | 0.2 | 0.001 | 1472 | 1 | 0.9 | 0.004 |
|  | *Methanocellaconradii* | 340 | 1 | 0.1 | 0.0008 | 733 | 1 | 0.4 | 0.002 |
|  | *Methanocellapaludicola SANAE* | 532 | 1 | 0.5 | 0.003 | 1375 | 1 | 0.8 | 0.004 |
| *Methanococcoides* | *Methanococcoidesmethylutens* | **677** | **1** | **0.2** | **0.002** | **215** | **1** | **0.1** | **0.0006** |
|  | *Methanococcoidesburtonii* | 261 | 1 | 0.09 | 0.0006 | 162 | 1 | 0.1 | 0.0005 |
|  | *Methanococcoidesvulcani* | **208** | **1** | **0.07** | **0.0005** | **70** | **1** | **0.04** | **0.0002** |
| *Methanohalobium* | *Methanohalobiumevestigatum* | 329 | 1 | 0.1 | 0.0008 | 185 | 1 | 0.1 | 0.0005 |
| *Methanohalophilus* | *Methanohalophilusmahii* | **159** | **1** | **0.05** | **0.0004** | **69** | **1** | **0.04** | **0.0002** |
|  | *Methanohalophilushalophilus* | **90** | **1** | **0.03** | **0.0002** | **30** | **1** | **0.02** | **0.00009** |
|  | *Methanohalophilusportucalensis* | **81** | **1** | **0.03** | **0.0002** | **40** | **1** | **0.02** | **0.0001** |
| *Methanolobus* | *Methanolobus profundi* | 430 | 1 | 0.1 | 0.001 | 199 | 1 | 0.1 | 0.0006 |
|  | *Methanolobuspsychrophilus* | 392 | 1 | 0.1 | 0.001 | 262 | 1 | 0.2 | 0.0008 |
|  | *Methanolobusvulcani* | **279** | **1** | **0.1** | **0.0007** | **134** | **1** | **0.08** | **0.0004** |
|  | *Methanolobustindarius* | **274** | **1** | **0.09** | **0.0007** | **139** | **1** | **0.08** | **0.0004** |
| *Methanosphaerula* | *Methanosphaerula palustris* | 386 | 1 | 0.1 | 0.0009 | 779 | 1 | 0.5 | 0.002 |
| *Methanospirillum* | *Methanospirillumhungatei* | 448 | 1 | 0.2 | 0.001 | 472 | 1 | 0.3 | 0.001 |
| *Methanothermus* | *Methanothermusfervidus* | **195** | **1** | **0.07** | **0.0005** | **105** | **1** | **0.06** | **0.0003** |
| *Methanotorris* | *Methanotorrisigneus* | **86** | **1** | **0.03** | **0.0002** | **46** | **1** | **0.03** | **0.0001** |
|  | *Methanotorrisformicicus* | **77** | **1** | **0.03** | **0.0002** | **39** | **1** | **0.02** | **0.0001** |

*Alphabets types in bold are most abundant in mangrove soil compared to non-mangrove soil.

**Table S9.** Relative abundance of nitrifying bacteria in Mangrove and non-Mangrove area

|  |  | **Sundarbans/Mangrove** | | | **Non-Mangrove** | | |
| --- | --- | --- | --- | --- | --- | --- | --- |
| **Genus name** | **Species name** | **No. of species** | **%Bacteria** | **%All** | **No. of species** | **%Bacteria** | **%All** |
| *Nitrobacter* | *N. hamburgensis* | 1 | 0.004 | 0.001 | 1 | 0.01 | 0.005 |
|  | *N. vulgaris* | 1 | 0.002 | 0.0004 | 1 | 0.006 | 0.002 |
|  | *N. winogradskyi* | 1 | 0.003 | 0.0007 | 1 | 0.006 | 0.002 |
|  | *Nitrobacter sp. Nb-311A* | 1 | 0.003 | 0.0008 | 1 | 0.008 | 0.003 |
| *Nitrococcus* | *Nitrococcusmobilis* | **1** | **0.03** | **0.008** | **1** | **0.01** | **0.004** |
| *Nitrosococcus* | *Nitrosococcusoceani* | **3** | **0.02** | **0.005** | **3** | **0.008** | **0.003** |
|  | *Nitrosococcushalophilus* | **1** | **0.05** | **0.01** | **1** | **0.01** | **0.005** |
|  | *Nitrosococcuswatsonii* | **1** | **0.01** | **0.003** | **1** | **0.005** | **0.002** |
| *Nitrosomonas* | *N. aestuarii* | **1** | **0.01** | **0.003** | **1** | **0.005** | **0.002** |
|  | *N. communis* | **1** | **0.009** | **0.002** | **1** | **0.007** | **0.003** |
|  | *N. europaea* | **1** | **0.005** | **0.001** | **1** | **0.003** | **0.001** |
|  | *N. eutropha* | **1** | **0.007** | **0.002** | **1** | **0.004** | **0.002** |
|  | *N. halophila* | **1** | **0.01** | **0.002** | **1** | **0.004** | **0.002** |
|  | *N. marina* | **1** | **0.02** | **0.004** | **1** | **0.004** | **0.002** |
|  | *N. nitrosa* | **1** | **0.007** | **0.002** | **1** | **0.004** | **0.001** |
|  | *N. oligotropha* | **1** | **0.003** | **0.0008** | **1** | **0.002** | **0.0007** |
|  | *N. ureae* | **1** | **0.007** | **0.002** | **1** | **0.004** | **0.001** |
|  | *N. mobilis* | **1** | **0.007** | **0.002** | **1** | **0.004** | **0.001** |
|  | *N. Cryotolerans* | **1** | **0.008** | **0.002** | **1** | **0.003** | **0.001** |
| *Nitrospira* | *N. moscoviensis* | **1** | **0.03** | **0.007** | **1** | **0.03** | **0.01** |
|  | *Nitrospira japonica* | **1** | **0.03** | **0.006** | **1** | **0.02** | **0.008** |
|  | *Nitrospiradefluvii* | **1** | **0.007** | **0.002** | **1** | **0.006** | **0.002** |
|  | *Nitrospira sp. ST-bin5* | **1** | **0.02** | **0.005** | **1** | **0.01** | **0.005** |
|  | *Nitrospira sp. SG-bin2* | **1** | **0.02** | **0.004** | **1** | **0.007** | **0.002** |
| *Nitrospina* | *Nitrospinagracilis* | **1** | **0.1** | **0.02** | **1** | **0.009** | **0.003** |
|  | *Nitrospina sp. SCGC_AAA799_C22* | **1** | **0.02** | **0.004** | **1** | **0.003** | **0.001** |
|  | *Nitrospina sp. SCGC_AAA799_A02* | **1** | **0.007** | **0.002** | **1** | **0.001** | **0.0005** |
|  | *Nitrospinagracilis 3/211* | **1** | **0.003** | **0.0007** | **1** | **0.0006** | **0.0002** |
| *Nitrosospira* | *Nitrosospiramultiformis* | 1 | 0.01 | 0.003 | 1 | 0.01 | 0.004 |
|  | *Nitrosospirabriensis* | 1 | 0.006 | 0.001 | 1 | 0.007 | 0.002 |
|  | *Nitrosospiralacus* | 1 | 0.003 | 0.0008 | 1 | 0.003 | 0.0009 |
|  | *Nitrosospira sp. 56-18* | **1** | **0.008** | **0.002** | **1** | **0.006** | **0.002** |
|  | *Nitrosospira sp. Nsp14* | 1 | 0.006 | 0.001 | 1 | 0.006 | 0.002 |
|  | *Nitrosospira sp. Nl5* | 1 | 0.005 | 0.001 | 1 | 0.005 | 0.002 |
|  | *Nitrosospira sp. NpAV* | 1 | 0.005 | 0.001 | 1 | 0.003 | 0.001 |
|  | *Nitrosospira sp. Nsp11* | **1** | **0.002** | **0.0005** | **1** | **0.0009** | **0.0003** |
|  | *Nitrosospira sp. Nsp13* | 1 | 0.001 | 0.0004 | 1 | 0.002 | 0.0008 |
|  | *Nitrosospira sp. Nsp1* | 1 | 0.001 | 0.0003 | 1 | 0.001 | 0.0004 |
|  | *Nitrosospira sp. Nsp18* | 1 | **0.001** | **0.0002** | **1** | **0.0009** | **0.0003** |
|  | *Nitrosospira sp. 40KI* | 1 | **0.00002** | **0.000005** | **0** | **0** | **0** |

*Alphabets types in bold are more abundant in mangrove than in non-mangrove soil.

**Table S10.** Salt tolerant bacteria identified in Mangrove and non-mangrove forest

|  |  | **Sundarbans/Mangrove** | | | **Non-Mangrove** | |  |
| --- | --- | --- | --- | --- | --- | --- | --- |
| **Sl. No.** | **Name of species** | **Total count** | **%Bacteria** | **%All** | **Total count** | **%Bacteria** | **%All** |
| 1 | *Alkalibacillushaloalkaliphilus* | **145** | **0.001** | **0.0004** | **110** | **0.0009** | **0.0003** |
| 2 | *Halomonasanticariensis* | **1152** | **0.01** | **0.003** | **577** | **0.005** | **0.002** |
| 3 | *Halomonaselongata* | **637** | **0.006** | **0.002** | **200** | **0.002** | **0.0006** |
| 4 | *Halomonasutahensis* | **1846** | **0.02** | **0.005** | **431** | **0.003** | **0.001** |
| 5 | *Halomonasmuralis* | 756 | 0.008 | 0.002 | 395 | 0.003 | 0.001 |
| 6 | *Halomonashalmophila* | **2** | **0.00002** | **0.000005** | 0 | 0 | 0 |
| 7 | *Halomonashalodenitrificans* | **409** | **0.004** | **0.001** | **143** | **0.001** | **0.0004** |
| 8 | *Halomonas sp. Marseille-P2426* | **1312** | **0.01** | **0.003** | **123** | **0.001** | **0.0004** |
| 9 | *Halomonasilicicola* | **733** | **0.007** | **0.002** | **372** | **0.003** | **0.001** |
| 10 | *Halomonas salina* | **787** | **0.008** | **0.002** | **269** | **0.002** | **0.0008** |
| 11 | *Halomonaskorlensis* | **698** | **0.007** | **0.002** | **482** | **0.004** | **0.001** |
| 12 | *Marinococcushalotolerans* | **49** | **0.0005** | **0.0001** | **32** | **0.0002** | **0.00009** |
| 13 | *Salinicoccus albus* | **125** | **0.001** | **0.0003** | **79** | **0.0006** | **0.0002** |
| 14 | *Salinicoccusqingdaonensis* | **55** | **0.0005** | **0.0001** | **47** | **0.0004** | **0.0001** |
| 15 | *Salinicoccus luteus* | **44** | **0.0004** | **0.0001** | **34** | **0.0003** | **0.0001** |
| 16 | *Salinivibriosocompensis* | **114** | **0.001** | **0.0003** | **22** | **0.0002** | **0.00006** |
| 17 | *Salinicoccushalodurans* | **57** | **0.0006** | **0.0001** | **57** | **0.0004** | **0.0002** |
| 18 | *Marinococcus luteus* | 35 | 0.0003 | 0.00009 | 74 | 0.0006 | 0.0002 |
| 19 | *Staphylococcus aureus* | 1665 | 0.02 | 0.004 | 59618 | 0.5 | 0.2 |
| 20 | *Actinopolyspora halophila* | 69 | 0.0007 | 0.0002 | 440 | 0.003 | 0.001 |
| 21 | *Salinicoccusalkaliphilus* | 103 | 0.001 | 0.0003 | 105 | 0.005 | 0.002 |
|  |  |  |  |  |  |  |  |
| **Sl. No.** | **Aerobic or facultatively anaerobic gram-negative moderately halophilic bacteria** | | | | | |  |
| 1 | *Salinivibriocosticola* | **1781** | **0.02** | **0.004** | **13** | **0.0001** | **0.00004** |
| 2 | *Arhodomonasaquaeolei* | 2948 | 0.03 | 0.007 | 1274 | 0.01 | 0.004 |
|  |  |  |  |  |  |  |  |
| **Sl. No.** | **Aerobic gram-positive moderately halophilic bacteria** | | |  |  |  |  |
| 1 | *Halobacillushalophilus* | 80 | 0.0008 | 0.0002 | 47 | 0.0004 | 0.0001 |
| 2 | *Halobacillusmangrovi* | **95** | **0.0009** | **0.0002** | **0** | **0** | **0** |
| 3 | *Halobacillustrueperi* | **39** | **0.0004** | **0.0001** | **18** | **0.0001** | **0.00005** |
| 4 | *Tetragenococcusmuriaticus* | **62** | **0.0006** | **0.0002** | **57** | **0.0004** | **0.0002** |
| 5 | *Tetragenococcushalophilus* | **76** | **0.0008** | **0.0002** | **56** | **0.0004** | **0.0002** |
| 6 | *Marinococcushalophilus* | **68** | **0.0007** | **0.0002** | **67** | **0.0005** | **0.0002** |
| 7 | *Salinicoccus roseus* | **44** | **0.0004** | **0.0001** | **44** | **0.0003** | **0.0001** |
| 8 | *Halobacillusdabanensis* | 83 | 0.0008 | 0.0002 | 103 | 0.0008 | 0.0003 |
| 9 | *Halobacillusmassiliensis* | 176 | 0.002 | 0.0004 | 199 | 0.002 | 0.0006 |
| 10 | *Halobacillushunanensis* | 130 | 0.001 | 0.0003 | 151 | 0.001 | 0.0004 |
| 11 | *Halobacilluskuroshimensis* | 120 | 0.001 | 0.0003 | 202 | 0.002 | 0.0006 |
| 12 | *Tetragenococcus solitarius* | **46** | **0.0005** | **0.0001** | **49** | **0.0004** | **0.0001** |
| 13 | *Halobacillussalinus* | **110** | **0.001** | **0.0003** | **120** | **0.0009** | **0.0003** |
|  |  |  |  |  |  |  |  |
| **Sl. No.** | **Moderately halophilic actinomycete species** | | |  |  |  |  |
| 1 | *Actinopolysporamortivallis* | 123 | 0.001 | 0.0003 | 535 | 0.004 | 0.002 |
| 2 | *Actinopolysporamzabensis* | 74 | 0.0007 | 0.0002 | 246 | 0.002 | 0.0007 |
| 3 | *Nocardiopsislucentensis* | 166 | 0.002 | 0.0004 | 483 | 0.004 | 0.001 |
| 4 | *Nocardiopsis halophila* | 33 | 0.0003 | 0.00008 | 128 | 0.001 | 0.0004 |
| 5 | *Nocardiopsisgilva* | 252 | 0.003 | 0.0006 | 899 | 0.007 | 0.003 |
| 6 | *Nocardiopsis salina* | 132 | 0.001 | 0.0003 | 543 | 0.004 | 0.002 |
| 7 | *Nocardiopsishalotolerans* | 68 | 0.0007 | 0.0002 | 438 | 0.003 | 0.001 |
| 8 | *Nocardiopsispotens* | 236 | 0.002 | 0.0006 | 1337 | 0.01 | 0.004 |

*Alphabets types in bold are most abundant in mangrove soil compared to non-mangrove soil

**Table S11.** Relative abundance of methanotrophs bacteria in Mangrove versus Non-Mangrove soils

|  |  | **Mangrove** | | | | **Non-Mangrove** | | | |
| --- | --- | --- | --- | --- | --- | --- | --- | --- | --- |
|  | **Species** | **total count** | **no. of species** | **% Bacteria** | **% All** | **total count** | **no. of species** | **% Bacteria** | **% All** |
| *Methylobacter* | *Methylobactertundripaludum* | **2001** | **1** | **0.02** | **0.005** | **1178** | **1** | **0.009** | **0.003** |
|  | *Methylobacterluteus* | **1463** | **1** | **0.01** | **0.004** | **1004** | **1** | **0.008** | **0.003** |
|  | *Methylobactermarinus* | **300** | **1** | **0.003** | **0.0007** | **165** | **1** | **0.001** | **0.0005** |
|  | *Methylobacterwhittenburyi* | **277** | **1** | **0.003** | **0.0007** | **154** | **1** | **0.001** | **0.0004** |
|  | *Methylobacter sp. BBA5.1* | **223** | **1** | **0.002** | **0.0005** | **144** | **1** | **0.001** | **0.0004** |
| *Methylococcus* | *Methylococcuscapsulatus* | **2269** | **1** | **0.02** | **0.006** | **1231** | **1** | **0.01** | **0.004** |
|  | *Methylococcuscapsulatus str. Bath* | **63** | **1** | **0.0006** | **0.0002** | **74** | **1** | **0.0006** | **0.0002** |
| *Methylophaga* | *Methylophagathiooxydans* | **27259** | **1** | **0.3** | **0.07** | **290** | **1** | **0.002** | **0.0008** |
|  | *Methylophagaaminisulfidivorans* | **4944** | **1** | **0.05** | **0.01** | **113** | **1** | **0.0009** | **0.0003** |
|  | *Methylophagalonarensis* | **4224** | **1** | **0.04** | **0.01** | **273** | **1** | **0.002** | **0.0008** |
|  | *Methylophagasulfidovorans* | **3918** | **1** | **0.04** | **0.01** | **102** | **1** | **0.0008** | **0.0003** |
|  | *Methylophagafrappieri* | **3743** | **1** | **0.04** | **0.009** | **241** | **1** | **0.002** | **0.0007** |
|  | *Methylophaganitratireducenticrescens* | **2709** | **1** | **0.03** | **0.007** | **213** | **1** | **0.002** | **0.0006** |
|  | *Methylophagamuralis* | **2634** | **1** | **0.03** | **0.006** | **215** | **1** | **0.002** | **0.0006** |
| *Methylophilus* | *Methylophilusmethylotrophus* | **132** | **1** | **0.001** | **0.0003** | **89** | **1** | **0.0007** | **0.0003** |
|  | *Methylophilus sp. TWE2* | **118** | **1** | **0.001** | **0.0003** | **104** | **1** | **0.0008** | **0.0003** |
|  | *Methylophilus sp. 5* | 144 | 1 | 0.001 | 0.0004 | 148 | 1 | 0.001 | 0.0004 |
|  | *Methylophilusrhizosphaerae* | 191 | 1 | 0.002 | 0.0005 | 204 | 1 | 0.002 | 0.0006 |
| *Methylovorus* | *Methylovorus sp. MM1* | **763** | **1** | **0.008** | **0.002** | **492** | **1** | **0.004** | **0.001** |
|  | *Methylovorusglucosotrophus* | **81** | **1** | **0.0008** | **0.0002** | **78** | **1** | **0.0006** | **0.0002** |
|  | *Methylovorus sp. MP688* | 49 | 1 | 0.0005 | 0.0001 | 60 | 1 | 0.0005 | 0.0002 |
| *Methylosarcina* | *Methylosarcinafibrata* | **1795** | **1** | **0.02** | **0.004** | **1290** | **1** | **0.01** | **0.004** |
|  | *Methylosarcinalacus* | **1317** | **1** | **0.01** | **0.003** | **909** | **1** | **0.007** | **0.003** |
| *Methylacidiphilum* | *Methylacidiphiluminfernorum* | 290 | 1 | 0.003 | 0.0007 | 523 | 1 | 0.004 | 0.002 |
|  | *Methylacidiphilumfumariolicum* | 102 | 1 | 0.001 | 0.0002 | 228 | 1 | 0.002 | 0.0007 |
|  | *Methylacidiphilumkamchatkense* | 87 | 1 | 0.0009 | 0.0002 | 162 | 1 | 0.001 | 0.0005 |
| *Methylibium* | *Methylibium sp. CF059* | 687 | 1 | 0.007 | 0.002 | 1771 | 1 | 0.01 | 0.005 |
|  | *Methylibium sp. NZG* | 495 | 1 | 0.005 | 0.001 | 1305 | 1 | 0.01 | 0.004 |
|  | *Methylibiumpetroleiphilum* | 149 | 1 | 0.001 | 0.0004 | 171 | 1 | 0.001 | 0.0005 |
| *Methylobacillus* | *Methylobacillus sp. MM2* | 1045 | 1 | 0.01 | 0.003 | 1232 | 1 | 0.01 | 0.004 |
|  | *Methylobacillusflagellatus* | 489 | 1 | 0.005 | 0.001 | 1085 | 1 | 0.008 | 0.003 |
|  | *Methylobacillusglycogenes* | 326 | 1 | 0.003 | 0.0008 | 3572 | 1 | 0.03 | 0.01 |
| *Methylobacterium* | *Methylobacteriumnodulans* | 1033 | 1 | 0.01 | 0.003 | 2368 | 1 | 0.02 | 0.007 |
|  | *Methylobacteriumaquaticum* | 534 | 1 | 0.005 | 0.001 | 1021 | 1 | 0.008 | 0.003 |
|  | *Methylobacteriumextorquens* | 523 | 1 | 0.005 | 0.001 | 742 | 1 | 0.006 | 0.002 |
|  | *Methylobacteriumpopuli* | 391 | 1 | 0.004 | 0.001 | 863 | 1 | 0.007 | 0.003 |
|  | *Methylobacteriummesophilicum* | 252 | 1 | 0.003 | 0.0006 | 737 | 1 | 0.006 | 0.002 |
| *Methylocapsa* | *Methylocapsapalsarum* | 557 | 1 | 0.006 | 0.001 | 1657 | 1 | 0.01 | 0.005 |
|  | *Methylocapsaacidiphila* | 513 | 1 | 0.005 | 0.001 | 1600 | 1 | 0.01 | 0.005 |
|  | *Methylocapsaaurea* | 471 | 1 | 0.005 | 0.001 | 1475 | 1 | 0.01 | 0.004 |
| *Methylocella* | *Methylocellasilvestris* | 540 | 1 | 0.005 | 0.001 | 1742 | 1 | 0.01 | 0.005 |
| *Methylocystis* | *Methylocystisbryophila* | 673 | 1 | 0.007 | 0.002 | 1685 | 1 | 0.01 | 0.005 |
|  | *Methylocystisparvus* | 466 | 1 | 0.005 | 0.001 | 1423 | 1 | 0.01 | 0.004 |
|  | *Methylocystisrosea* | 172 | 1 | 0.002 | 0.0004 | 417 | 1 | 0.003 | 0.001 |
| *Methylosinus* | *Methylosinus sp. PW1* | 210 | 1 | 0.002 | 0.0005 | 546 | 1 | 0.004 | 0.002 |
|  | *Methylosinus sp. LW4* | 193 | 1 | 0.002 | 0.0005 | 393 | 1 | 0.003 | 0.001 |
|  | *Methylosinus sp. R-45379* | 89 | 1 | 0.0009 | 0.0002 | 176 | 1 | 0.001 | 0.0005 |
|  | *Methylosinus sp. LW3* | 63 | 1 | 0.0006 | 0.0002 | 263 | 1 | 0.002 | 0.0008 |
|  | *Methylosinustrichosporium* | 15 | 1 | 0.0001 | 0.00004 | 36 | 1 | 0.0003 | 0.0001 |
| *Methylotenera* | *Methyloteneraversatilis* | 669 | 1 | 0.007 | 0.002 | 737 | 1 | 0.006 | 0.002 |
|  | *Methyloteneramobilis* | 441 | 1 | 0.004 | 0.001 | 454 | 1 | 0.004 | 0.001 |
|  | *Methylotenera sp. G11* | 403 | 1 | 0.004 | 0.001 | 1046 | 1 | 0.008 | 0.003 |
|  | *Methylotenera sp. L2L1* | 138 | 1 | 0.001 | 0.0003 | 147 | 1 | 0.001 | 0.0004 |

*Alphabets types in bold are most abundant in mangrove soil compared to non-mangrove soil.

**Table S12.** Relative abundance of pathogenic bacteria in Mangrove and non-Mangrove area

|  | **Sundarbans/Mangrove(M)** | | | **Non-Mangrove area(N)** | | |  | **Differences(M-N)** | | |
| --- | --- | --- | --- | --- | --- | --- | --- | --- | --- | --- |
| **Genus/name** | **Total count** | **%Bacteria** | **%All** | **Total count** | **%Bacteria** | **%All** |  | **Total count** | **%Bacteria** | **%All** |
| *Pseudomonas aeruginosa* | **43** | **0.04** | **0.009** | **38** | **0.02** | **0.008** |  | **5** | **0.02** | **0.001** |
| *Vibrio cholerae* | **24** | **0.02** | **0.004** | **5** | **0.004** | **0.001** |  | **19** | **0.016** | **0.003** |
| *Clostridium botulinum* | **19** | **0.02** | **0.004** | **11** | **0.006** | **0.002** |  | **8** | **0.014** | **0.002** |
| *Mycoplasma capricolum* | **2** | **0.0006** | **0.0001** | **1** | **0.00002** | **9E-06** |  | **1** | **0.00058** | **0.000091** |
| *Leptospirainterrogans* | **10** | **0.003** | **0.0007** | **5** | **0.0009** | **0.0003** |  | **5** | **0.0021** | **0.0004** |
| *Francisellanoatunensis* | **3** | **0.002** | **0.0005** | **9** | **0.00007** | **0.00003** |  | **-6** | **0.00193** | **0.00047** |
| *Enterobacter cloacae* | **13** | **0.01** | **0.003** | **8** | **0.003** | **0.001** |  | **5** | **0.007** | **0.002** |
| *Klebsiella pneumoniae* | **25** | **0.01** | **0.003** | **26** | **0.006** | **0.002** |  | **-1** | **0.004** | **0.001** |
| *Edwardsiellatarda* | **3** | **0.002** | **0.0006** | **1** | **0.0006** | **0.0002** |  | **2** | **0.0014** | **0.0004** |
| *Citrobacterfreundii* | **1** | **0.002** | **0.0005** | **1** | **0.0005** | **0.0002** |  | **0** | **0.0015** | **0.0003** |
| *Proteus mirabilis* | **2** | **0.002** | **0.0005** | **2** | **0.001** | **0.0004** |  | **0** | **0.001** | **0.0001** |
| *Morganellamorganii* | **1** | **0.003** | **0.0008** | **1** | **0.0007** | **0.0003** |  | **0** | **0.0023** | **0.0005** |
| *Providencia stuartii* | **2** | **0.004** | **0.0009** | **2** | **0.001** | **0.0004** |  | **0** | **0.003** | **0.0005** |
| *Campylobacter fetus* | **2** | **0.008** | **0.002** | **1** | **0.001** | **0.0004** |  | **1** | **0.007** | **0.0016** |
| *Brucella abortus* | **2** | **0.002** | **0.0005** | **2** | **0.0003** | **0.0001** |  | **0** | **0.0017** | **0.0004** |
| *Salmonella enterica* | **87** | **0.02** | **0.006** | **48** | **0.02** | **0.006** |  | **39** | **0** | **0** |
| *Escherichia coli* | **175** | **0.05** | **0.01** | **90** | **0.03** | **0.01** |  | **85** | **0.02** | **0** |
| *Bacillus cereus* | **1** | **0.02** | **0.004** | **25** | **0.01** | **0.004** |  | **-24** | **0.01** | **0** |
| *Clostridium tetani* | **1** | **0.002** | **0.0004** | **2** | **0.0007** | **0.0003** |  | **-1** | **0.0013** | **0.0001** |
| *Pasteurellamultocida* | **4** | **0.005** | **0.001** | **5** | **0.001** | **0.0004** |  | **-1** | **0.004** | **0.0006** |
| *Mycobacterium tuberculosis* | 21 | 0.01 | 0.003 | 37 | 0.04 | 0.01 |  | -16 | -0.03 | -0.007 |
| *Acinetobacter baumannii* | 44 | 0.02 | 0.004 | 29 | 0.1 | 0.05 |  | 15 | -0.08 | -0.046 |
| *Staphylococcus aureus* | 23 | 0.02 | 0.004 | 16 | 0.5 | 0.2 |  | 7 | -0.48 | -0.196 |
| *Shigellasonnei* | 1 | 0.003 | 0.0007 | 1 | 0.004 | 0.001 |  | 0 | -0.001 | -0.0003 |
| *Brucella suis* | 2 | 0.0004 | 0.0001 | 2 | 0.0005 | 0.0002 |  | 0 | -0.0001 | -0.0001 |
| *Brucella melitensis* | 4 | 0.0002 | 0.00006 | 3 | 0.0006 | 0.0002 |  | 1 | -0.0004 | -0.00014 |
| *Salmonella enterica subsp. enterica serovar Typhi* | 1 | 0.0005 | 0.0001 | 44 | 0.006 | 0.002 |  | -43 | -0.0055 | -0.0019 |

*Alphabets types in bold are most abundant in mangrove soil compared to non-mangrove soil

**Table S13.** Relative abundance of bioremediation bacteria in Mangrove and non-Mangrove area

|  |  | **Sundarbans/Mangrove(M)** | | | **Non-Mangrove(N)** | | | **Differences (M-N)** | | |
| --- | --- | --- | --- | --- | --- | --- | --- | --- | --- | --- |
| **Sl. No.** | **Name of species** | **Total count** | **% Bacteria** | **%All** | **Total count** | **% Bacteria** | **%All** | **Total count** | **%Bacteria** | **%All** |
| 1 | *Pseudomonas alcaligenes* | **1082** | **0.01** | **0.003** | **710** | **0.006** | **0.002** | **372** | **0.004** | **0.001** |
| 2 | *Pseudomonas mendocina* | **9851** | **0.1** | **0.02** | **318** | **0.002** | **0.0009** | **9533** | **0.098** | **0.0191** |
| 3 | *Pseudomonas pseudoalcaligenes* | **551** | **0.005** | **0.001** | **362** | **0.003** | **0.001** | **189** | **0.002** | **0** |
| 4 | *Pseudomonas resinovorans* | **957** | **0.01** | **0.002** | **745** | **0.006** | **0.002** | **212** | **0.004** | **0** |
| 5 | *Pseudomonas veronii* | **123** | **0.001** | **0.0003** | **96** | **0.0007** | **0.0003** | **27** | **0.0003** | **0** |
| 6 | *Pseudomonas putida* | **1774** | **0.02** | **0.004** | **1233** | **0.01** | **0.004** | **541** | **0.01** | **0** |
| 7 | *Alcanivoraxborkumensis* | **91** | **0.0009** | **0.0002** | **4** | **0.00003** | **0.00001** | **87** | **0.00087** | **0.0002** |
| 8 | *Desulfovibrio vulgaris* | **1176** | **0.01** | **0.003** | **770** | **0.006** | **0.002** | **406** | **0.004** | **0.001** |
| 9 | *Desulfovibriodesulfuricans* | **1704** | **0.02** | **0.004** | **848** | **0.007** | **0.002** | **856** | **0.013** | **0.002** |
| 10 | *Escherichia coli* | **5103** | **0.05** | **0.01** | **4240** | **0.03** | **0.01** | **863** | **0.02** | **0** |
| 11 | *Paracoccusdenitrificans* | **292** | **0.003** | **0.0007** | **178** | **0.001** | **0.0005** | **114** | **0.002** | **0.0002** |
| 12 | *Shewanellaputrefaciens* | **107** | **0.001** | **0.0003** | **42** | **0.0003** | **0.0001** | **65** | **0.0007** | **0.0002** |
| 13 | *Geobactersulfurreducens* | 1428 | 0.01 | 0.003 | 1270 | 0.01 | 0.004 | 158 | 0 | -0.001 |
| 14 | *Geobactermetallireducens* | 2333 | 0.02 | 0.006 | 2452 | 0.02 | 0.007 | -119 | 0 | -0.001 |
| 15 | *Methylibiumpetroleiphilum* | 149 | 0.001 | 0.0004 | 171 | 0.001 | 0.0005 | -22 | 0 | -1E-04 |
| 16 | *Nitrosomonaseuropaea* | 505 | 0.005 | 0.001 | 351 | 0.003 | 0.001 | 154 | 0.002 | 0 |
| 17 | *Nitrobacterhamburgensis* | 408 | 0.004 | 0.001 | 1635 | 0.01 | 0.005 | -1227 | -0.006 | -0.004 |
| 18 | *Rhodoferaxferrireducens* | 692 | 0.007 | 0.002 | 1383 | 0.01 | 0.004 | -691 | -0.003 | -0.002 |
| 19 | *Pseudomonas stutzeri* | 2588 | 0.03 | 0.006 | 6506 | 0.05 | 0.02 | -3918 | -0.02 | -0.014 |
| 20 | *Dechloromonasaromatica* | 562 | 0.006 | 0.001 | 650 | 0.005 | 0.002 | -88 | 0.001 | -0.001 |
| 21 | *Deinococcusradiodurans* | 201 | 0.002 | 0.0005 | 442 | 0.003 | 0.001 | -241 | -0.001 | -5E-04 |

*Alphabets types in bold are most abundant in mangrove soil compared to non-mangrove soil.

**Table S14.** Relative abundance of probiotics bacteria in Mangrove and non-Mangrove area

|  | **Mangrove (M)** | |  |  | **Non -Mangrove (N)** |  | **Difference(M-N)** | |  |
| --- | --- | --- | --- | --- | --- | --- | --- | --- | --- |
|  | **Total count** | **% Bacteria** | **% All** | **Total count** | **% Bacteria** | **% All** | **Total count** | **% Bacteria** | **% All** |
| *Vibrio mediterranei* | **24** | **0.0002** | **0.00006** | **10** | **0.00008** | **0.00003** | **14** | **0.00012** | **0.00003** |
| *Vibrio fluvialis* | **884** | **0.009** | **0.002** | **39** | **0.0003** | **0.0001** | **845** | **0.0087** | **0.0019** |
| *Vibrio harveyi* | **325** | **0.1** | **0.03** | **57** | **0.0004** | **0.0002** | **268** | **0.0996** | **0.0298** |
| *Vibrio alginolyticus* | **910** | **0.1** | **0.03** | **123** | **0.01** | **0.004** | **787** | **0.09** | **0.026** |
| *Bacillus Clausii* | **95** | **0.0009** | **0.0002** | **86** | **0.0007** | **0.0002** | **9** | **0.0002** | **0** |
| *Bacillus subtilis* | **9234** | **0.09** | **0.02** | **185** | **0.001** | **0.0005** | **9049** | **0.089** | **0.0195** |
| *Bacillus cereus* | **1516** | **0.02** | **0.004** | **1256** | **0.01** | **0.004** | **260** | **0.01** | **0** |
| *Bacillus mycoides* | **151** | **0.002** | **0.0004** | **88** | **0.0007** | **0.0003** | **63** | **0.0013** | **0.0001** |
| *Bacillus pumilus* | **299** | **0.003** | **0.0007** | **164** | **0.001** | **0.0005** | **135** | **0.002** | **0.0002** |
| *Bacillus Licheniformis* | **13977** | **0.1** | **0.03** | **142** | **0.001** | **0.0004** | **13835** | **0.099** | **0.0296** |
| *Lactobacillus Curvatus* | **59** | **0.0006** | **0.0001** | **35** | **0.0003** | **0.0001** | **24** | **0.0003** | **0** |
| *Lactobacillus helveticus* | **31** | **0.0003** | **0.00008** | **29** | **0.0002** | **0.00008** | **2** | **0.0001** | **0** |
| *Lactobacillus buchneri* | **31** | **0.0003** | **0.00008** | **13** | **0.0001** | **0.00004** | **18** | **0.0002** | **0.00004** |
| *Lactobacillus Gasseri* | **44** | **0.0004** | **0.0001** | **22** | **0.0002** | **0.00006** | **22** | **0.0002** | **0.00004** |
| *Lactobacillus rhamnosus* | **84** | **0.0008** | **0.0002** | **57** | **0.0004** | **0.0002** | **27** | **0.0004** | **0** |
| *Lactobacillus plantarum* | **140** | **0.001** | **0.0003** | **117** | **0.0009** | **0.0003** | **23** | **0.0001** | **0** |
| *Bifidobacterium bifidum* | **49** | **0.02** | **0.006** | **131** | **0.001** | **0.0004** | **-82** | **0.019** | **0.0056** |
| *Bifidobacterium longum* | **105** | **0.02** | **0.006** | **152** | **0.001** | **0.0004** | **-47** | **0.019** | **0.0056** |
| *Micrococcus luteus* | **83** | **0.003** | **0.0009** | **173** | **0.001** | **0.0005** | **-90** | **0.002** | **0.0004** |
| *RoseobacterlitoralisOch 149* | **7** | **0.00007** | **0.00002** | **8** | **0.00006** | **0.00002** | **-1** | **1E-05** | **0** |
| *Lactobacillus delbrueckii* | **63** | **0.0006** | **0.0002** | **44** | **0.0003** | **0.0001** | **19** | **0.0003** | **0.0001** |
| *Lactobacillus salivarius* | **155** | **0.002** | **0.0004** | **90** | **0.0007** | **0.0003** | **65** | **0.0013** | **0.0001** |
| *Lactobacillus johnsonii* | **26** | **0.0003** | **0.00006** | **15** | **0.0001** | **0.00004** | **11** | **0.0002** | **0.00002** |
| *Lactobacillus reuteri* | **129** | **0.001** | **0.0003** | **83** | **0.0006** | **0.0002** | **46** | **0.0004** | **0.0001** |
| *Bifidobacterium adolescentis* | **131** | **0.02** | **0.006** | **110** | **0.0009** | **0.0003** | **21** | **0.0191** | **0.0057** |
| *Shewanellacolwelliana* | **489** | **0.005** | **0.001** | **90** | **0.0007** | **0.0003** | **399** | **0.0043** | **0.0007** |
| *Shewanellaputrefaciens* | **107** | **0.001** | **0.0003** | **42** | **0.0003** | **0.0001** | **65** | **0.0007** | **0.0002** |
| *Pediococcuspentosaceus* | **46** | **0.0005** | **0.0001** | **26** | **0.0002** | **0.00008** | **20** | **0.0003** | **0.00002** |
| *Pediococcusacidilactici* | **72** | **0.004** | **0.0009** | **59** | **0.0005** | **0.0002** | **13** | **0.0035** | **0.0007** |
| *Enterococcus durans* | **26950** | **0.3** | **0.07** | **178** | **0.03** | **0.01** | **26772** | **0.27** | **0.06** |
| *Streptococcus thermophilus* | **36** | **0.05** | **0.01** | **33** | **0.0003** | **0.0001** | **3** | **0.0497** | **0.0099** |
| *Leuconostocmesenteroides* | **120** | **0.005** | **0.001** | **74** | **0.0006** | **0.0002** | **46** | **0.0044** | **0.0008** |
| *Aeromonashydrophila* | **589** | **0.006** | **0.001** | **1** | **0.000008** | **0.000003** | **588** | **0.005992** | **0.000997** |
| *Paenibacilluspolymyxa* | **471** | **0.005** | **0.001** | **296** | **0.002** | **0.0009** | **175** | **0.003** | **0.0001** |
| *Lactococcuslactis* | **389** | **0.3** | **0.07** | **251** | **0.002** | **0.02** | **138** | **0.298** | **0.05** |
| *Carnobacteriumdivergens* | **104** | **0.001** | **0.0003** | **87** | **0.0007** | **0.0003** | **17** | **0.0003** | **0** |
| *Carnobacteriummaltaromaticum* | **172** | **0.009** | **0.002** | **94** | **0.0007** | **0.0003** | **78** | **0.0083** | **0.0017** |
| *Roseobacterlitoralis* | **421** | **0.004** | **0.001** | **8** | **0.00006** | **0.00002** | **413** | **0.00394** | **0.00098** |
| *Roseobacterdenitrificans* | **305** | **0.03** | **0.008** | **91** | **0.0007** | **0.0003** | **214** | **0.0293** | **0.0077** |
| *RoseobacterdenitrificansOCh 114* | **9** | **0.00009** | **0.00002** | **7** | **0.00005** | **0.00002** | **2** | **0.00004** | **0** |
| *Vagococcusteuberi* | **23** | **0.0002** | **0.00006** | **18** | **0.0001** | **0.00005** | **5** | **0.0001** | **0.00001** |
| *Oenococcusalcoholitolerans* | **40** | **0.0004** | **0.0001** | **38** | **0.0003** | **0.0001** | **2** | **0.0001** | **0** |
| *Lactobacillus crispatus* | **108** | **0.001** | **0.0003** | **117** | **0.0009** | **0.0003** | **-9** | **0.0001** | **0** |
| *Bacillus circulans* | **96** | **0.001** | **0.0002** | **101** | **0.0008** | **0.0003** | **-5** | **0.0002** | **-0.0001** |
| *Lactobacillus casei* | 30 | 0.002 | 0.0004 | 29 | 0.002 | 0.0006 | 1 | 0 | -0.0002 |
| *Lactobacillus paracasei* | 56 | 0.0006 | 0.0001 | 41 | 0.002 | 0.0006 | 15 | -0.0014 | -0.0005 |
| *Lactobacillus acidophilus* | 10 | 0.0001 | 0.00002 | 13 | 0.0001 | 0.00004 | -3 | 0 | -0.00002 |
| *Bacillus megaterium* | 213 | 0.002 | 0.0005 | 193 | 0.002 | 0.0006 | 20 | 0 | -1.000E-04 |
| *Bifidobacterium animalis* | 44 | 0.0004 | 0.0001 | 113 | 0.0009 | 0.0003 | -69 | -0.0005 | -0.0002 |
| *Bifidobacterium breve* | 58 | 0.0006 | 0.0001 | 108 | 0.0008 | 0.0003 | -50 | -0.0002 | -0.0002 |
| *Pseudomonas fluorescens* | 2122 | 0.02 | 0.005 | 1992 | 0.02 | 0.006 | 130 | 0 | -0.001 |
| *Enterococcus faecium* | 306 | 0.003 | 0.0007 | 323 | 0.03 | 0.01 | -17 | -0.027 | -0.0093 |
| *Oenococcusoeni* | 110 | 0.001 | 0.0003 | 88 | 0.01 | 0.005 | 22 | -0.009 | -0.0047 |
| *Oenococcuskitaharae* | 35 | 0.0003 | 0.00009 | 46 | 0.0004 | 0.0001 | -11 | -0.0001 | -0.00001 |
| *Bacillus coagulans* | 346 | 0.003 | 0.0008 | 375 | 0.003 | 0.001 | -29 | 0 | -0.0002 |
| *Lactobacillus brevis* | 112 | 0.001 | 0.0003 | 190 | 0.001 | 0.0006 | -78 | 0 | -0.0003 |
| *Lactobacillus fermentum* | 104 | 0.001 | 0.0003 | 103 | 0.07 | 0.03 | 1 | -0.069 | -0.0297 |

*Alphabets types in bold are most abundant in mangrove soil compared to non-mangrove soil.

**Table S15.** Relative abundance of nitrifying bacteria in Mangrove and non-Mangrove area

|  |  | **Sundarbans/Mangrove** | | | **Non-Mangrove** | | |
| --- | --- | --- | --- | --- | --- | --- | --- |
| **Genus name** | **Species name** | **No. of species** | **%Bacteria** | **%All** | **No. of species** | **%Bacteria** | **%All** |
| *Nitrobacter* | *N. hamburgensis* | 1 | 0.004 | 0.001 | 1 | 0.01 | 0.005 |
|  | *N. vulgaris* | 1 | 0.002 | 0.0004 | 1 | 0.006 | 0.002 |
|  | *N. winogradskyi* | 1 | 0.003 | 0.0007 | 1 | 0.006 | 0.002 |
|  | *Nitrobacter sp. Nb-311A* | 1 | 0.003 | 0.0008 | 1 | 0.008 | 0.003 |
| *Nitrococcus* | *Nitrococcusmobilis* | **1** | **0.03** | **0.008** | **1** | **0.01** | **0.004** |
| *Nitrosococcus* | *Nitrosococcusoceani* | **3** | **0.02** | **0.005** | **3** | **0.008** | **0.003** |
|  | *Nitrosococcushalophilus* | **1** | **0.05** | **0.01** | **1** | **0.01** | **0.005** |
|  | *Nitrosococcuswatsonii* | **1** | **0.01** | **0.003** | **1** | **0.005** | **0.002** |
| *Nitrosomonas* | *N. aestuarii* | **1** | **0.01** | **0.003** | **1** | **0.005** | **0.002** |
|  | *N. communis* | **1** | **0.009** | **0.002** | **1** | **0.007** | **0.003** |
|  | *N. europaea* | **1** | **0.005** | **0.001** | **1** | **0.003** | **0.001** |
|  | *N. eutropha* | **1** | **0.007** | **0.002** | **1** | **0.004** | **0.002** |
|  | *N. halophila* | **1** | **0.01** | **0.002** | **1** | **0.004** | **0.002** |
|  | *N. marina* | **1** | **0.02** | **0.004** | **1** | **0.004** | **0.002** |
|  | *N. nitrosa* | **1** | **0.007** | **0.002** | **1** | **0.004** | **0.001** |
|  | *N. oligotropha* | **1** | **0.003** | **0.0008** | **1** | **0.002** | **0.0007** |
|  | *N. ureae* | **1** | **0.007** | **0.002** | **1** | **0.004** | **0.001** |
|  | *N. mobilis* | **1** | **0.007** | **0.002** | **1** | **0.004** | **0.001** |
|  | *N. Cryotolerans* | **1** | **0.008** | **0.002** | **1** | **0.003** | **0.001** |
| *Nitrospira* | *N. moscoviensis* | **1** | **0.03** | **0.007** | **1** | **0.03** | **0.01** |
|  | *Nitrospira japonica* | **1** | **0.03** | **0.006** | **1** | **0.02** | **0.008** |
|  | *Nitrospiradefluvii* | **1** | **0.007** | **0.002** | **1** | **0.006** | **0.002** |
|  | *Nitrospira sp. ST-bin5* | **1** | **0.02** | **0.005** | **1** | **0.01** | **0.005** |
|  | *Nitrospira sp. SG-bin2* | **1** | **0.02** | **0.004** | **1** | **0.007** | **0.002** |
| *Nitrospina* | *Nitrospinagracilis* | **1** | **0.1** | **0.02** | **1** | **0.009** | **0.003** |
|  | *Nitrospina sp. SCGC_AAA799_C22* | **1** | **0.02** | **0.004** | **1** | **0.003** | **0.001** |
|  | *Nitrospina sp. SCGC_AAA799_A02* | **1** | **0.007** | **0.002** | **1** | **0.001** | **0.0005** |
|  | *Nitrospinagracilis 3/211* | **1** | **0.003** | **0.0007** | **1** | **0.0006** | **0.0002** |
| *Nitrosospira* | *Nitrosospiramultiformis* | 1 | 0.01 | 0.003 | 1 | 0.01 | 0.004 |
|  | *Nitrosospirabriensis* | 1 | 0.006 | 0.001 | 1 | 0.007 | 0.002 |
|  | *Nitrosospiralacus* | 1 | 0.003 | 0.0008 | 1 | 0.003 | 0.0009 |
|  | *Nitrosospira sp. 56-18* | **1** | **0.008** | **0.002** | **1** | **0.006** | **0.002** |
|  | *Nitrosospira sp. Nsp14* | 1 | 0.006 | 0.001 | 1 | 0.006 | 0.002 |
|  | *Nitrosospira sp. Nl5* | 1 | 0.005 | 0.001 | 1 | 0.005 | 0.002 |
|  | *Nitrosospira sp. NpAV* | 1 | 0.005 | 0.001 | 1 | 0.003 | 0.001 |
|  | *Nitrosospira sp. Nsp11* | **1** | **0.002** | **0.0005** | **1** | **0.0009** | **0.0003** |
|  | *Nitrosospira sp. Nsp13* | 1 | 0.001 | 0.0004 | 1 | 0.002 | 0.0008 |
|  | *Nitrosospira sp. Nsp1* | 1 | 0.001 | 0.0003 | 1 | 0.001 | 0.0004 |
|  | *Nitrosospira sp. Nsp18* | 1 | **0.001** | **0.0002** | **1** | **0.0009** | **0.0003** |
|  | *Nitrosospira sp. 40KI* | 1 | **0.00002** | **0.000005** | **0** | **0** | **0** |

*Alphabets types in bold are most abundant in mangrove soil compared to non-mangrove soil.


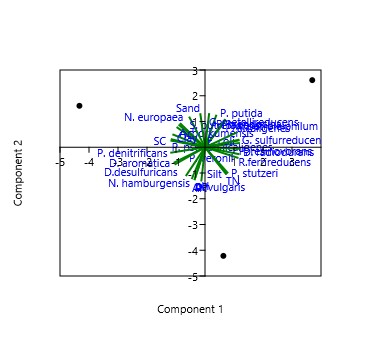

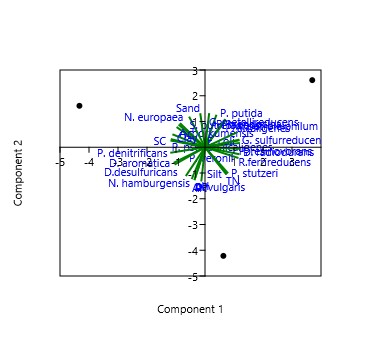


Bioremediation bacteria

A

B

Methanogenic bacteria


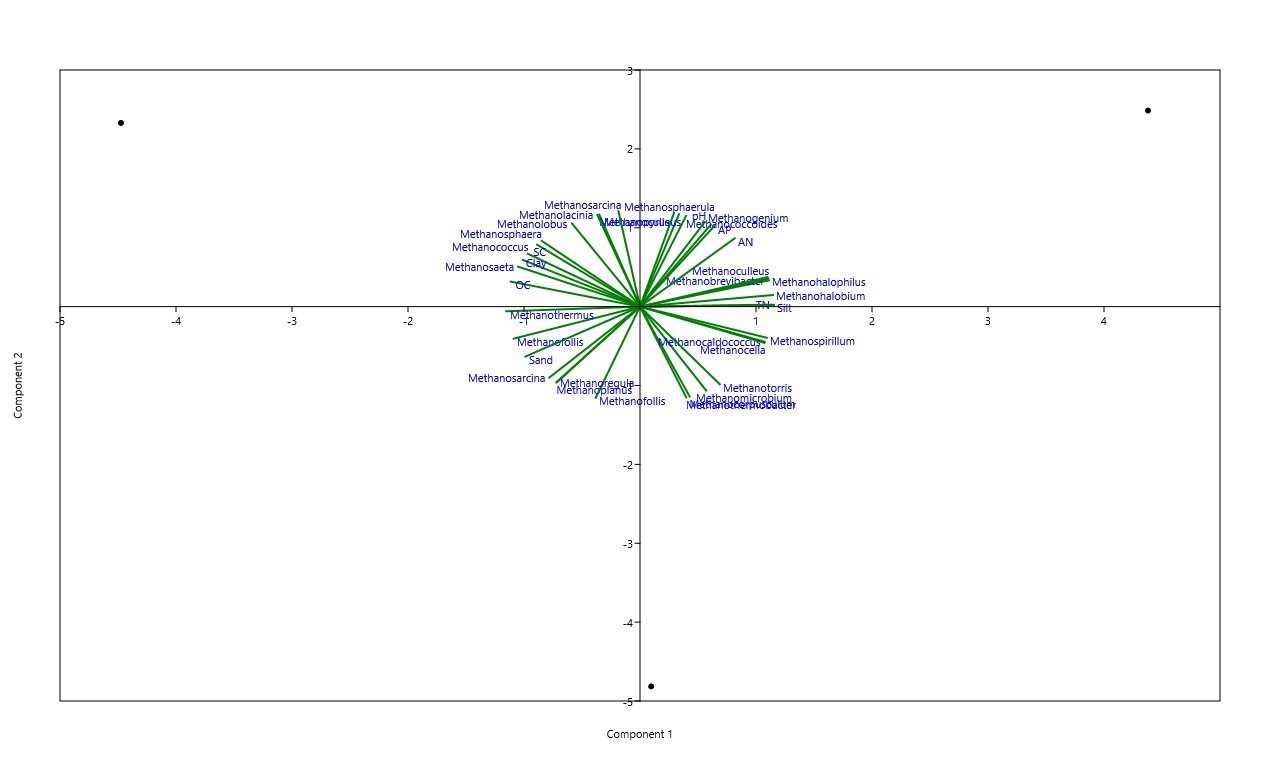

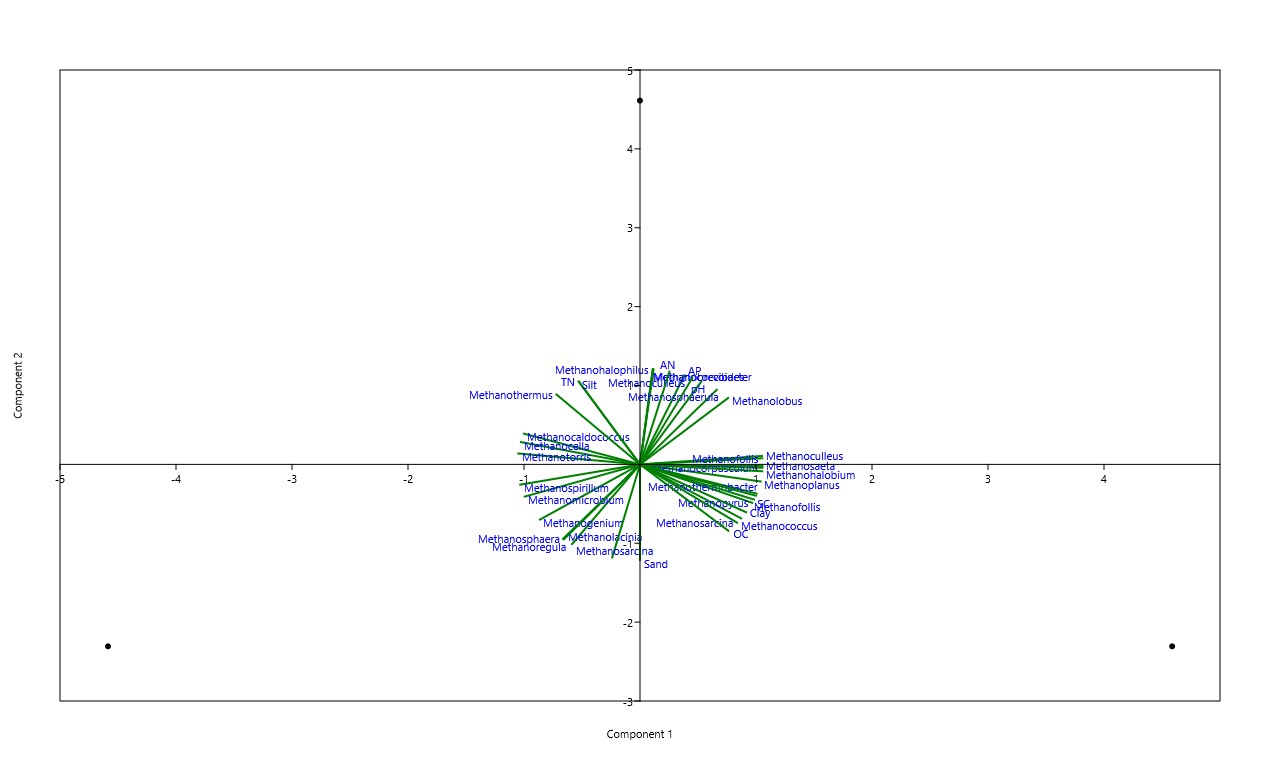


A

B

Methanotrophs bacteria


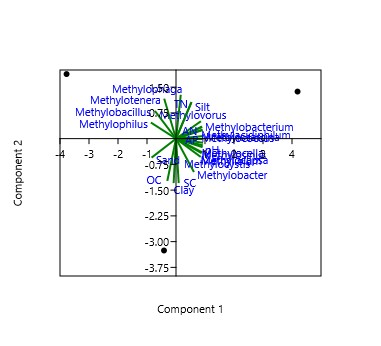

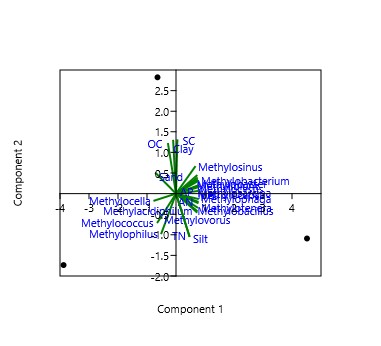


A

B

Pathogenic bacteria

A

B


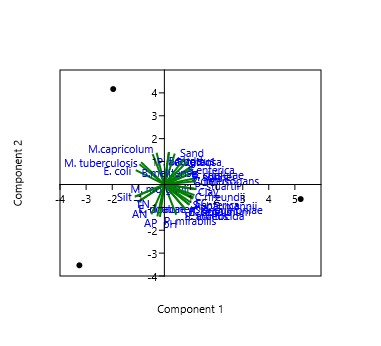

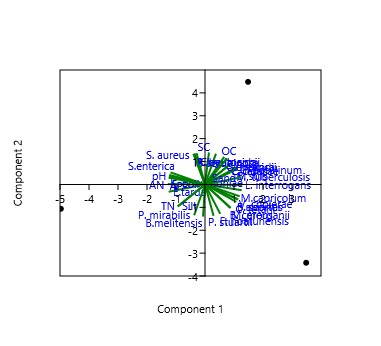


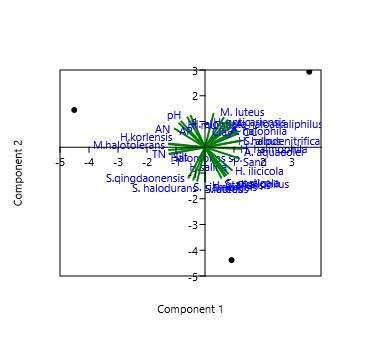

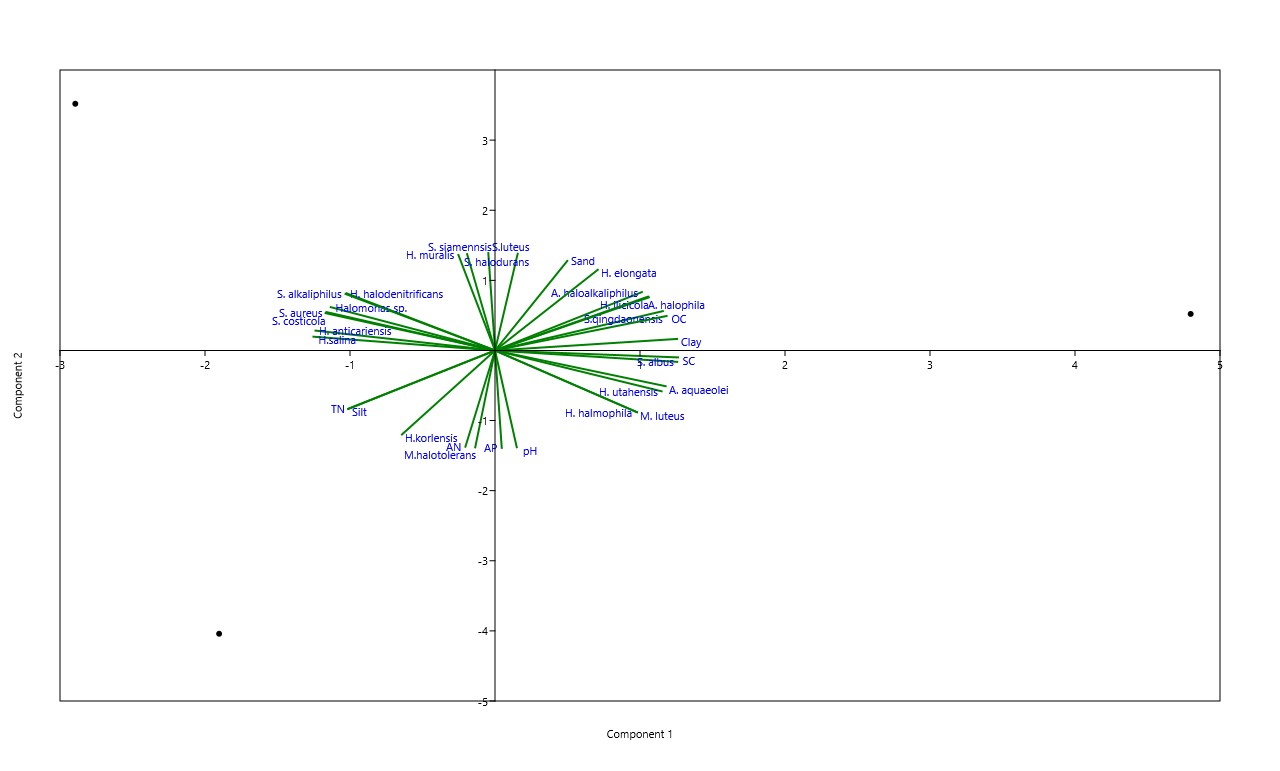


Salt tolerant bacteria

A

B

**Figure S1.** PCA plot showing the differences among sampling sites based on sediment quality parameters and microbial abundance Bioremediation, Methanogenic, Methanotrophs, Pathogenic and Salt tolerant bacteria. (A) Samples collected from non-mangrove; (B) Salt collected from mangrove sediments.
